# Probing gene function in *Candida albicans* wild-type strains by Cas9-facilitated one-step integration of two dominant selection markers: a systematic analysis of recombination events at the target locus

**DOI:** 10.1128/msphere.00388-24

**Published:** 2024-06-28

**Authors:** Bernardo Ramírez-Zavala, Anna Hoffmann, Ines Krüger, Sonja Schwanfelder, Katherine S. Barker, P. David Rogers, Joachim Morschhäuser

**Affiliations:** 1 Institute of Molecular Infection Biology, University of Würzburg, Würzburg, Germany; 2 Department of Pharmacy and Pharmaceutical Sciences, St. Jude Children’s Research Hospital, Memphis, Tennessee, USA; University of Georgia, Athens, Georgia, USA

**Keywords:** *Candida albicans*, gene deletion, CRISPR-Cas9, loss of heterozygosity, homozygous mutants

## Abstract

The adaptation of gene deletion methods based on the CRISPR-Cas9 system has facilitated the genetic manipulation of the pathogenic yeast *Candida albicans*, because homozygous mutants of this diploid fungus can now be generated in a single step, allowing the rapid screening of candidate genes for their involvement in a phenotype of interest. However, the Cas9-mediated double-strand breaks at the target site may result in an undesired loss of heterozygosity (LOH) on the affected chromosome and cause phenotypic alterations that are not related to the function of the investigated gene. In our present study, we harnessed Cas9-facilitated gene deletion to probe a set of genes that are constitutively overexpressed in strains containing hyperactive forms of the transcription factor Mrr1 for a possible contribution to the fluconazole resistance of such strains. To this aim, we used gene deletion cassettes containing two different dominant selection markers, *caSAT1* and *HygB*, which confer resistance to nourseothricin and hygromycin, respectively, for simultaneous genomic integration in a single step, hypothesizing that this would minimize undesired LOH events at the target locus. We found that selection for resistance to both nourseothricin and hygromycin strongly increased the proportion of homozygous deletion mutants among the transformants compared with selection on media containing only one of the antibiotics, but it did not avoid undesired LOH events. Our results demonstrate that LOH on the target chromosome is a significant problem when using Cas9 for the generation of *C. albicans* gene deletion mutants, which demands a thorough examination of recombination events at the target site.

**IMPORTANCE:**

*Candida albicans* is one of the medically most important fungi and a model organism to study fungal pathogenicity. Investigating gene function in this diploid yeast has been facilitated by the adaptation of gene deletion methods based on the bacterial CRISPR-Cas9 system, because they enable the generation of homozygous mutants in a single step. We found that, in addition to increasing the efficiency of gene replacement by selection markers, the Cas9-mediated double-strand breaks also result in frequent loss of heterozygosity on the same chromosome, even when two different selection markers were independently integrated into the two alleles of the target gene. Since loss of heterozygosity for other genes can result in phenotypic alterations that are not caused by the absence of the target gene, these findings show that it is important to thoroughly analyze recombination events at the target locus when using Cas9 to generate gene deletion mutants in *C. albicans*.

## INTRODUCTION

Infections by the pathogenic yeast *Candida albicans* are commonly treated with fluconazole, a well-tolerated drug that inhibits ergosterol biosynthesis and thereby growth of the fungus. *C. albicans* can develop fluconazole resistance by various mechanisms, which often involve the overexpression of genes encoding multidrug efflux pumps, the ATP-binding cassette transporters Cdr1 and Cdr2 and the major facilitator Mdr1 (1, 2). The constitutive overexpression of these genes in fluconazole-resistant isolates is caused by gain-of-function (GOF) mutations in their main transcriptional regulators, Tac1, which controls *CDR1* and *CDR2* expression, and Mrr1, which regulates *MDR1* expression (3–11). Of note, the upregulation of other target genes of these transcription factors also contributes to Tac1- and Mrr1-mediated drug resistance. For example, the deletion of *PDR16*, which encodes a phosphatidylinositol transfer protein, in a strain with a Tac1 GOF mutation decreased its resistance, and forced *PDR16* overexpression in a susceptible strain resulted in increased fluconazole resistance (12). Likewise, an activated form of Mrr1 still conferred increased fluconazole resistance, albeit at a reduced level, when *MDR1* was deleted, demonstrating that other Mrr1 target genes are involved in the resistance phenotype (13). Transcriptional profiling of strains with Mrr1 GOF mutations identified genes that are regulated by Mrr1 (9, 13, 14). However, which of these genes contribute to fluconazole resistance has not been revealed so far. The deletion of orf19.7306, which encodes a putative aldo-keto reductase that was originally found by a proteomic analysis to be upregulated in *MDR1*-overexpressing strains, did not increase fluconazole susceptibility (15). Similarly, the deletion of *ALS1* and orf19.7042 (a gene of unknown function) did not reduce fluconazole resistance in a strain with a hyperactive Mrr1 (16). To our knowledge, no further efforts have been undertaken to establish which genes besides *MDR1* promote Mrr1-mediated fluconazole resistance.

The development of gene deletion methods based on the bacterial CRISPR-Cas9 system has facilitated the generation of specific knock-out mutants in *C. albicans* and other *Candida* species (17–29). The Cas9-mediated double-strand break at the target locus strongly increases the specific integration of selection markers in several non-*albicans Candida* species and other organisms. In *C. albicans*, homologous recombination is very efficient, and the vast majority of transformants exhibit a designed gene replacement without the help of Cas9. However, transformation efficiencies normally are too low to enable the simultaneous replacement of both alleles of a target gene in a single step in this diploid yeast, and the generation of homozygous deletion mutants traditionally required the sequential replacement of the two alleles with a recyclable marker or two different selection markers. This has changed with CRISPR-Cas9-based methods, since the double-strand breaks introduced by Cas9 must be repaired on both chromosomes, resulting in the inactivation of both alleles in many transformants. Furthermore, the requirement of a single instead of two sequential transformations also reduces the risk of stress-induced unspecific genomic alterations. A potential caveat that is not usually considered is that the deletion of both target gene alleles in some homozygous mutants may not have occurred by independent insertions of the selection marker but involved a single insertion into one chromosome, which then served as a repair template for the double-strand break on the homologous chromosome. This can result in loss of heterozygosity (LOH) for extended regions on this chromosome and, consequently, effects on the phenotype of the mutants that are not caused by the deletion of the target gene but by loss of allelic variants of other genes, especially when only one allele is functional (4, 30–48). In fact, it has recently been shown that CRISPR-Cas9-based gene editing strongly increased the frequency of LOH in *Candida parapsilosis*, especially on the target chromosome (49).

The ability to generate homozygous mutants in a single step enables a more rapid testing of candidate genes for their involvement in a specific phenotype. Most CRISPR-Cas9-based gene deletion methods use vectors containing the genes for the Cas9 endonuclease and a gene-specific single guide RNA, which are introduced into *C. albicans* cells together with the gene deletion cassette containing the selection marker. Instead of generating these expression constructs, one can also use a ribonucleoprotein (RNP) complex consisting of purified Cas9 enzyme and a custom-synthesized guide RNA (gRNA, a complex of a gene-specific crRNA and a universal tracrRNA) together with the gene deletion cassette containing the selection marker. The latter strategy was established for several genetically less tractable haploid *Candida* species and has recently also been used in *C. albicans* (17, 18, 20). In our present study, we used this approach to assess the contribution of previously identified Mrr1 target genes to fluconazole resistance of strains containing GOF mutations in this transcription factor. We hypothesized that the use of two different selection markers for Cas9-facilitated, separate replacement of the target gene alleles by the deletion cassettes reduces the occurrence of undesired LOH events on the target chromosome. For this purpose, we used gene deletion cassettes containing the *caSAT1* or the *HygB* marker, which confer resistance to nourseothricin and hygromycin, respectively, and are useful for the genetic manipulation of *C. albicans* wild-type strains (50, 51). We undertook a detailed analysis of the different gene deletion mutants and compared integration events in transformants after selection for resistance against one or both antibiotics.

## RESULTS

### Deletion of *GRP2* using PCR-amplified selection markers with short homology regions

**Fig 1:**
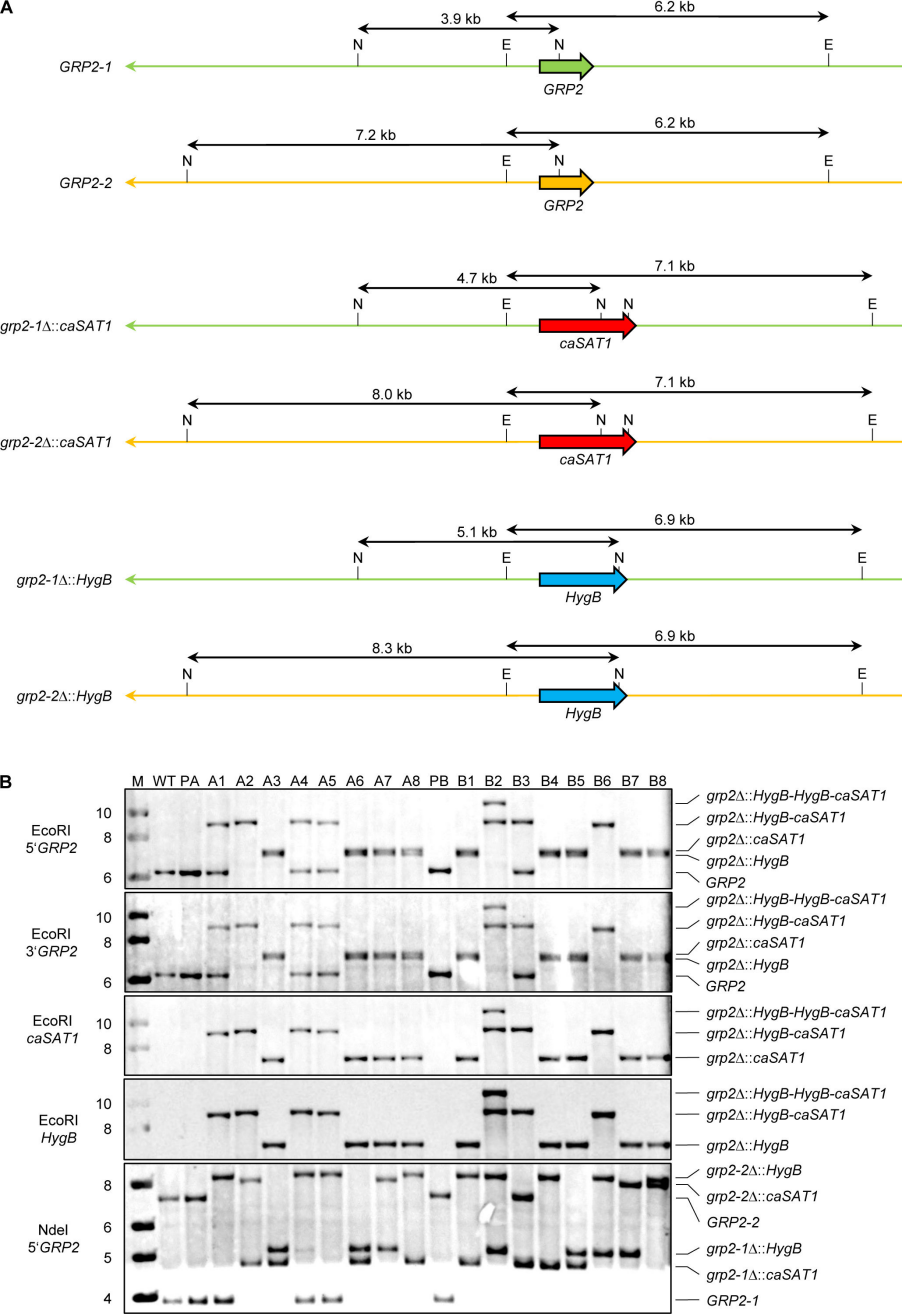
Deletion of *GRP2* using the *caSAT1* and *HygB* selection markers. (**A**) Structure of the *GRP2* locus in the wild type and mutants containing the *caSAT1* and *HygB* selection markers in either of the two *GRP2* alleles. Arrows on the lines representing the chromosomes point toward the telomere. The locations of diagnostic NdeI (N) and EcoRI (E) sites and the sizes of corresponding fragments are shown. (**B**) Southern hybridizations of EcoRI- or NdeI-digested genomic DNA of the wild-type strain SC5314 (WT), the parental strains SCMRR1R34A (PA) and SCMRR1R34B (PB), and nourseothricin- and hygromycin-resistant transformants A1 to A8 and B1 to B8, respectively, with the probes specified on the left. The identities of the hybridizing fragments are indicated on the right side of the blots. M, size markers (in kb).

**Fig 2:**
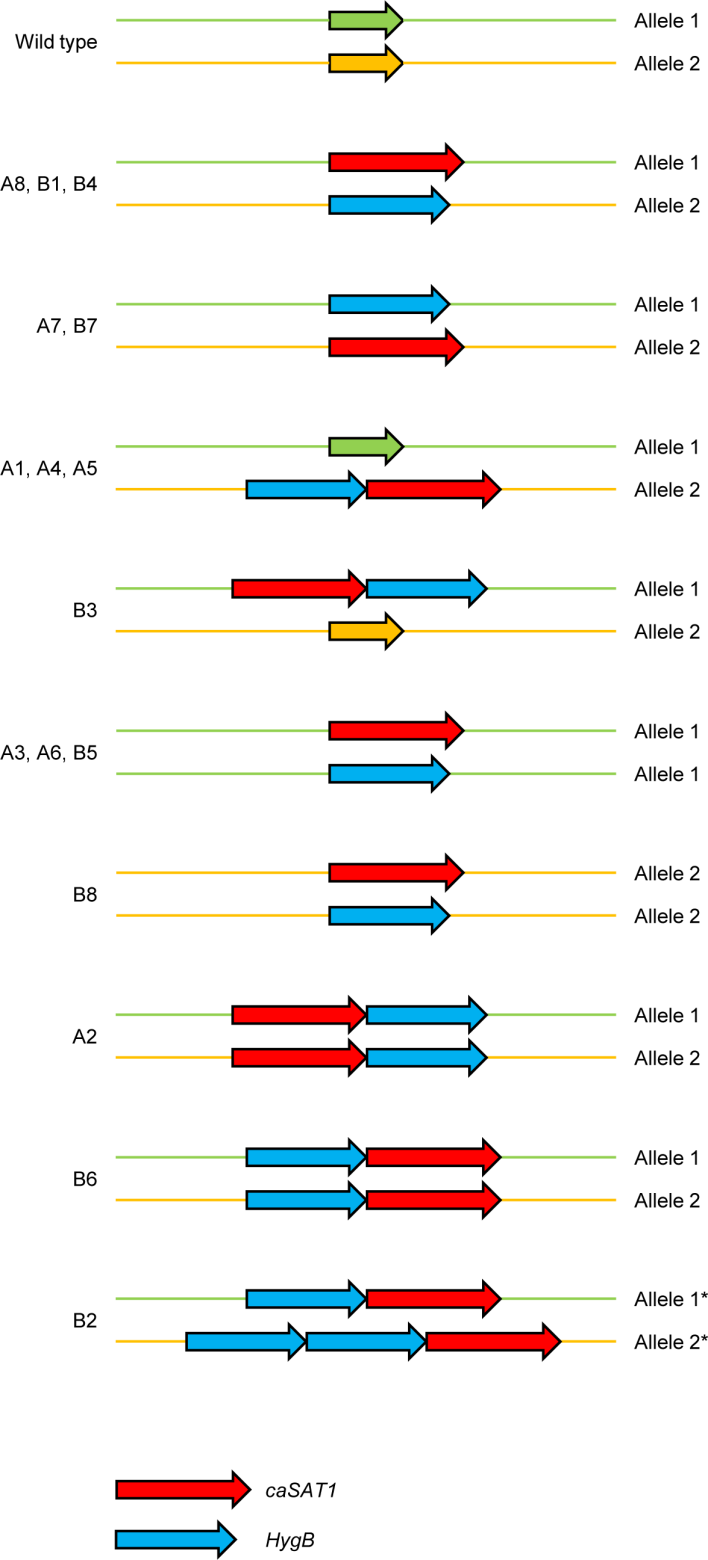
Genetic structure of *grp2*Δ mutants generated with *caSAT1* and *HygB* markers flanked by short homology regions. LOH in clones A3, A6, B5, and B8 is indicated by the same color of the flanking sequences, although we did not investigate the extent of the LOH. ^*^Or vice versa.

We chose *GRP2*, which is strongly upregulated in *C. albicans* strains containing GOF mutations in *MRR1* (13), as the first target for gene deletion with two different selection markers. The quickest way to obtain a gene deletion cassette is the amplification of a selection marker by PCR with oligonucleotides in which upstream and downstream sequences of the target gene are incorporated. Since the promoter (from the *ACT1* gene) and downstream sequences (from the *URA3* gene) of the *caSAT1* and *HygB* selection markers are identical, both selection markers could be amplified with the same pair of oligonucleotides. Equal amounts of the so-generated *GRP2* deletion cassettes were mixed with Cas9 enzyme and *GRP2*-specific gRNA and used to transform two independently generated derivatives of the *C. albicans* wild-type strain SC5314 that contained a GOF mutation in both *MRR1* alleles (see Materials and Methods for details). We selected 16 nourseothricin- and hygromycin-resistant transformants (eight from parent strain A and eight from parent strain B) from plates containing both antibiotics for Southern hybridization analysis. The expected outcome after the integration of the *caSAT1* and *HygB* selection markers into either of the two *GRP2* alleles is illustrated in Fig. 1A. Hybridization of EcoRI-digested genomic DNA with a probe from the *GRP2* upstream region demonstrated that 12 transformants had lost both *GRP2* alleles, whereas four transformants (clones A1, A4, A5, and B3) had retained one of the wild-type *GRP2* alleles (Fig. 1B, top panel). In addition to the wild-type fragment, these clones showed new bands that were larger than expected and corresponded to a tandem integration of both selection markers into one of the two *GRP2* alleles, which was confirmed by rehybridization of the blot with probes from the *GRP2* downstream region and with *caSAT1*- and *HygB*-specific probes (Fig. 1B, middle panels). The two *GRP2* alleles in strain SC5314 can be distinguished by an upstream NdeI restriction site polymorphism, and hybridization of NdeI-digested DNA showed that clones A1, A4, and A5 had integrated *HygB*, followed by *caSAT1*, in *GRP2* allele 2, while clone B3 contained *caSAT1*, followed by *HygB*, in *GRP2* allele 1 (Fig. 1B, bottom panel, see also Fig. 2 for an illustration of all integration events).

Of the 12 homozygous mutants, nine exhibited the hybridization pattern expected after integration of the *caSAT1* marker into one *GRP2* allele and integration of the *HygB* marker into the other (clones A3, A6, A7, A8, B1, B4, B5, B7, and B8; Fig. 1B, top panel). In contrast, clones A2 and B6 contained tandem integrations of *caSAT1* and *HygB* in both *GRP2* alleles, and clone B2 contained both markers in one allele and even three selection markers (two *HygB* copies and one *caSAT1* copy) in the other. PCRs with primers specific for each selection marker and a primer binding within the *GRP2* downstream region showed that in the latter case *caSAT1* followed two copies of *HygB*.

To better understand how the tandem integrations had occurred, we amplified the region between the *HygB* marker and the *caSAT1* marker from clones A1, A4, A5, B2, and B6 with specific primers and sequenced the PCR products. This analysis showed that the tandem integrations contained short deletions of variable sizes (ranging from 7 to 317 bp) in one of the two repair templates, which in two cases extended into the *HygB* downstream region (37 bp deleted in clone A5) or the *caSAT1* upstream region (227 bp deleted in clone B6) (Fig. S1).

Despite the fact that the two selection markers were independently integrated as single copies into either of the two *GRP2* alleles in nine transformants, examination of the NdeI hybridization patterns showed that only five of these were indeed correct, clones A8, B1, and B4 containing *caSAT1* in allele 1 and *HygB* allele 2 and clones A7 and B7 exhibiting the reverse pattern (Fig. 1B, bottom panel, and Fig. 2). In contrast, clones A3, A6, and B5 contained the allele-specific upstream NdeI site now on both chromosomes, while in clone B8 this NdeI site was not present anymore, demonstrating that the integration of the two markers was accompanied by an undesired LOH event in four of these nine homozygous mutants (Fig. 1B, bottom panel, and Fig. 2).

### Deletion of *GRP2* using selection markers with long homology regions

To investigate if the frequency of correct marker insertion could be improved by using longer homology regions, we generated deletion cassettes in which the *caSAT1* and *HygB* markers were flanked by ca. 0.5 kb of *GRP2* upstream and downstream sequences (identical for both markers). The two parental strains were transformed with equal amounts of the two deletion cassettes, either with or without Cas9 and the *GRP2*-specific gRNA, which allowed us to compare the transformation efficiencies. Aliquots of the cell suspensions were spread on plates containing either nourseothricin, hygromycin, or both antibiotics. As can be seen in Fig. S2, the inclusion of Cas9/gRNA strongly increased the transformation efficiency (more than we expected, which prevented precise colony counting even on the plates on which only 2% of the original cell suspension was plated).

We picked 24 transformants (usually 12 from parent strain A and 12 from parent strain B) for each condition for Southern hybridization analysis, the results of which are summarized in Table S1 (Southern blots are presented in Fig. S3). Of the 24 clones that were transformed without Cas9/gRNA and selected for nourseothricin resistance, 23 were heterozygous mutants that retained one of the wild-type *GRP2* alleles and contained a single copy of the *caSAT1* marker correctly integrated in the other allele. One clone (A7) still contained both wild-type *GRP2* alleles and had ectopically integrated multiple copies of *caSAT1* and *HygB* (Fig. S3A). Similarly, all 24 clones that were transformed without Cas9/gRNA and selected for hygromycin resistance were heterozygous mutants that retained one of the wild-type *GRP2* alleles. Of these, 22 clones had correctly inserted *HygB* in *GRP2* allele 1 or 2, while two clones (A2 and B12) contained multiple integrations of both markers in allele 2 (Fig. S3B). Somewhat surprisingly, we also obtained colonies on plates with both antibiotics when the Cas9/gRNA complex was not included in the transformation mixture (in this case, only 11 transformants were recovered from parent B, and 13 transformants were picked for parent A). However, none of the analyzed clones exhibited the designed gene replacements (Fig. S3C). Twenty-three clones were heterozygous mutants and retained a wild-type *GRP2* allele, 16 of which had correctly inserted *caSAT1* at the target locus. Two of these (A2 and B9) exhibited an additional band that corresponded to the integration of *HygB* in allele 2, possibly due to a recombination event in a subpopulation that gave rise to a mixed clone. The other 14 clones did not contain *HygB* and apparently had acquired hygromycin resistance by a different mechanism. The remaining seven heterozygous mutants contained multiple copies of both selection markers in one of the *GRP2* alleles. The single apparently homozygous mutant (A8) exhibited bands expected after the integration of *HygB* in allele 1 and *caSAT1* in allele 2, but also additional weak bands corresponding to wild-type allele 2 and integration of *caSAT1* in allele 1, again pointing to a mixed clone. In summary (and not surprisingly), no correct homozygous mutants could be obtained in a single transformation without the use of Cas9/gRNA even after selection for both resistance markers.

Of the 24 analyzed clones that were transformed in the presence of Cas9/gRNA and selected on nourseothricin plates, 13 were heterozygous mutants that retained one wild-type *GRP2* allele and had correctly inserted *caSAT1* in the other allele (Fig. S3D). Three further heterozygous mutants were mixed clones (due to the high colony density on the selection plate, the possibility of contamination by a different clone cannot be excluded in some cases). In addition to wild-type allele 1, clone A2 also contained integrations of *caSAT1* in both *GRP2* alleles. Clone A9 in addition to wild-type allele 1 and *caSAT1* integration in allele 2 exhibited weaker bands corresponding to wild-type allele 2 and *caSAT1* integration in allele 1. In clone B12, *caSAT1* was integrated into both alleles, but the signal for allele 1 was weaker, and some wild-type allele 1 was still present in the population. The other eight clones were homozygous mutants that had lost both wild-type *GRP2* alleles. Seven of them contained the *caSAT1* marker in both alleles, and one (A1) contained single integrations of *caSAT1* and *HygB* at the target locus. However, in three clones, the simultaneous deletion of both alleles was accompanied by an undesired LOH event. Clones A7 and B2 contained the diagnostic upstream NdeI site (which is located outside of the cloned flanking sequence of the deletion cassettes) on both chromosomes, whereas clone A1, despite the independent integration of both markers into the two *GRP2* alleles, had lost the upstream NdeI site on both chromosomes.

A similar result was obtained for transformants that were selected on hygromycin plates (Fig. S3E). Fourteen of the 24 analyzed clones were heterozygous mutants and contained *HygB* in one of the *GRP2* alleles. In clone A1, this was accompanied by an LOH event (NdeI site on both chromosomes), while A4 was a mixed clone that exhibited bands corresponding to wild-type allele 1, integration of *caSAT1* in allele 2, and integration of *HygB* in both alleles. The other 10 clones were homozygous *grp2*Δ mutants in which the *HygB* marker was correctly integrated at the target locus on both chromosomes without an apparent LOH event, except for clone B8 in which a subpopulation had the NdeI site upstream of allele 1 on both chromosomes.

All 24 transformants that were selected for resistance to both antibiotics were homozygous *grp2*Δ mutants (Fig. S3F). Fourteen of these had correctly inserted *caSAT1* in one allele and *HygB* in the other. Six other clones also contained independent integrations of the two selection markers into either of the two alleles but exhibited an undesired LOH at the target locus; clones A3, A7, A9, A12, and B10 had lost the NdeI site upstream of allele 1, and clone A2 contained the NdeI site on both chromosomes. Clones B1, B3, and B6 were mixed clones with additional bands (see Table S1 for details). Clone B4 showed weak hybridization signals from which the integration of the *HygB* marker could not be deduced, but this was not further examined.

In summary, the analysis of the *grp2*Δ mutants obtained with the different approaches showed the following: The inclusion of Cas9/gRNA with the deletion cassettes (in which case they served as repair templates for the Cas9-mediated double-strand breaks) enormously increased the transformation efficiency. As expected, it also enabled the isolation of homozygous deletion mutants after a single transformation when selecting for one resistance marker (8 out of 24 tested nourseothricin-resistant transformants and 10 out of 24 tested hygromycin-resistant transformants). Selection for both resistance markers strongly increased the frequency of homozygous mutants (all 24 tested transformants had lost both *GRP2* alleles). However, the simultaneous integration of selection markers into both *GRP2* alleles was frequently accompanied by recombination events between the two homologous chromosomes (presumably induced by the Cas9-mediated double-strand breaks) that resulted in an undesired LOH in neighboring regions, even when the *caSAT1* and *HygB* markers were independently integrated into the two *GRP2* alleles. Since the use of longer flanking sequences instead of short homology regions in the deletion cassettes improved the recovery of correct homozygous *grp2*Δ mutants (58% vs 31%), we decided to use this strategy and assess its efficiency for the deletion of other Mrr1 target genes. In all cases, the two parental strains were transformed with deletion cassettes containing the *caSAT1* and *HygB* selection markers and the RNP complex containing Cas9 and a gene-specific gRNA. Because of the high number of transformants obtained when including Cas9/gRNA, higher dilutions of the cell suspensions were plated to facilitate the isolation of individual clones for genetic analysis.

### Analysis of *glx3*Δ mutants

The two *GLX3* alleles of strain SC5314 can be distinguished by an upstream SpeI restriction site polymorphism (see Fig. S4A), and Southern hybridization analysis showed that 21 of the 24 tested nourseothricin-resistant transformants were heterozygous mutants that contained *caSAT1* in one of the *GLX3* alleles, which in one case (A3) was accompanied by an LOH event (Fig. S4B; Table S2). Two of the three homozygous deletion mutants (A6 and B9) contained *caSAT1* in both alleles, which in A6 came with LOH at the SpeI site, and one (A9) contained *caSAT1* in allele 1 and *HygB* in allele 2. Similarly, 21 of the 24 analyzed clones from the hygromycin plate were heterozygous mutants containing *HygB* in one of the *GLX3* alleles; two of these (B6 and B9) were mixed clones in which a subpopulation contained *HygB* also in the other allele. Of the three homozygous mutants, one (A3) contained *caSAT1* in allele 1 and *HygB* in allele 2; the other two (A12 and B7) contained *HygB* in both alleles, but A12 exhibited LOH in a subpopulation and B7 displayed LOH in the *GLX3* downstream region, as explained below for the other transformants.

As in the case of the *GRP2* mutants, selection for resistance to both antibiotics strongly increased the frequency of homozygous deletion mutants; all 24 analyzed clones had lost both *GLX3* copies and contained *caSAT1* in one allele and *HygB* in the other (Fig. S4B; Table S2). However, the majority of the mutants exhibited undesired LOH events. Clones A1, A7, A9, B2, and B8 contained the upstream SpeI site on both chromosomes, and similar LOH events had occurred in subpopulations of clones A5, A10, A11, B3, and B10. Since the SpeI site is located between *GLX3* and the centromere, and LOH events caused by break-induced replication are more likely to occur between a double-strand break and the telomere, we also tested for LOH events at a SalI site downstream of *GLX3* allele 1, which could be conveniently monitored by hybridization of PstI/SalI-digested genomic DNA with a *GLX3* downstream probe (see Fig. S4A). This analysis uncovered LOH in additional clones (A6, A8, B6, B9, and B11; Fig. S4C). In summary, only nine of the 24 tested homozygous mutants obtained after selection for resistance to both nourseothricin and hygromycin had specifically replaced both *GLX3* alleles by the *caSAT1* and *HygB* markers without an LOH event at the target locus (Table S2).

### Analysis of *oye23*Δ mutants

The two *OYE23* alleles in strain SC5314 can be distinguished by a ClaI site that is present only in the coding region of allele 1 (Fig. S5A). Since this polymorphism is lost in homozygous deletion mutants, transformants were also analyzed for LOH in the *PKH3* gene, which is located 243 kb upstream of *OYE23* on the same chromosome arm, 61 kb from the telomere (an HhaI site is present only in *PKH3* allele 1). Only three of the 24 analyzed nourseothricin-resistant clones were homozygous mutants in which both *OYE23* alleles were replaced by *caSAT1* (Fig. S5B and C; Table S3). Two of these (A11 and B10) contained subpopulations with LOH at the *PKH3* locus, and the other (B1) also contained cells that had integrated *HygB* instead of *caSAT1* in one of the *OYE23* alleles. The remaining clones were heterozygous mutants, although in two of them (A6 and B6) a subpopulation had lost the second wild-type *OYE23* allele. Similarly, three of the 24 tested clones from the hygromycin plate were homozygous mutants, two of which contained *HygB* in both alleles without (A3) or with (B9) LOH at the *PKH3* locus; the third homozygous mutant (B12) contained *HygB* in one allele and *caSAT1* in the other (Fig. S5B and C; Table S3). Of the 21 heterozygous mutants, one (B8) had lost both *OYE23* alleles in the majority of cells. Selection for resistance to both antibiotics resulted in 22 homozygous mutants containing *caSAT1* in one allele and *HygB* in the other; the other two analyzed clones contained a tandem integration of *caSAT1* and *HygB* in allele 1 (A2) or allele 2 (A10). LOH at the *PKH3* locus was observed only in four of the homozygous mutants (A5, A11, B6, and B8), but since *PKH3* is located far away from *GLX3*, LOH events in the vicinity of the target gene in some of the other mutants cannot be excluded (Fig. S5B and C; Table S3).

### Analysis of *oye32*Δ mutants

The two *OYE32* alleles in strain SC5314 can be distinguished by an AgeI site upstream of allele 2 (Fig. S6A). Of the 22 analyzed nourseothricin-resistant transformants, only one (B2) was a homozygous mutant in which both *OYE32* alleles were replaced by *caSAT1*; all others retained one *OYE32* copy, albeit in three cases (A2, A8, and A12) only in a subpopulation (Fig. S6B; Table S4). Of the 24 analyzed clones from hygromycin plates, four (A6, A11, B3, and B7) had correctly integrated *HygB* in both *OYE32* alleles. Two other homozygous mutants (B5 and B10) contained subpopulations with LOH at the AgeI site, and an additional homozygous mutant (A7) was a mixed clone that contained *HygB* in allele 1 and either *HygB* or *caSAT1* in allele 2, with LOH in a subpopulation. The other transformants retained a wild-type *OYE32* copy at least in a subpopulation (Fig. S6C; Table S4). Of the 24 analyzed clones that were selected for resistance to both nourseothricin and hygromycin, three (A7, B5, and B6) had retained a wild-type *OYE32* allele and contained tandem integrations of *caSAT1* and *HygB* in the other allele; B11 was a mixed clone with a tandem integration of *caSAT1* and *HygB* in allele 2 and *caSAT1* integration in allele 1 in a subpopulation. Of the 20 homozygous mutants, 18 contained single integrations of *caSAT1* in one allele and *HygB* in the other, while two of them (A8 and B1) contained a tandem integration of both markers in allele 1 and either *caSAT1* or *HygB* in allele 2. Only 11 of the 18 homozygous mutants with single marker integrations were correct mutants, and in seven of them (A1, A4, A6, A11, B3, B8, and B10), LOH at the AgeI site had occurred at least in a subpopulation (Fig. S6D; Table S4).

## DISCUSSION

An initial goal of our study was to elucidate which of the Mrr1 target genes that are strongly upregulated in strains containing GOF mutations in this transcription factor contribute to the acquired fluconazole resistance of such strains. Deletion of none of the four candidate genes that were tested in the present report resulted in increased fluconazole susceptibility [minimal inhibitory concentration (MIC) was the same for the parental strains and all mutants (16 µg/mL), a 32-fold increase compared to the wild-type strain SC5314 (0.5 µg/mL)]. While additional Mrr1 target genes can be tested in the future, we focused the study on the insights gained from the detailed analysis of the mutants generated so far by Cas9-facilitated gene deletion and the use of two different selection markers.

Our results confirm that the combination of gene-specific deletion cassettes with purified Cas9 enzyme and a gene-specific gRNA is a convenient way to generate homozygous deletion mutants in a single step in *C. albicans* (20). The simplest method to produce the deletion cassettes is marker amplification by PCR with primers containing short homology regions to the target gene, which can be done with the same primer pair for both selection markers used in our study, *caSAT1* and *HygB*, because they contain identical upstream and downstream sequences. We tested this strategy for the deletion of the *GRP2* gene. Despite the presence of only short homology regions in the deletion cassettes, marker integration had occurred at the target locus in all 16 transformants that were analyzed, due to the necessity to repair the Cas9-mediated double-strand breaks. Nevertheless, not all transformants that had acquired resistance to both nourseothricin and hygromycin were homozygous deletion mutants, because in some cases the two selection markers were integrated in tandem in only one of the two alleles. This had occurred via homologous recombination of the genomic target sequences with the upstream flanking sequence of one marker and the downstream flanking sequence of the other marker and additional nonhomologous end joining of the two markers. While only five of the 16 tested transformants exhibited the designed replacement of both *GRP2* alleles by single integrations of *caSAT1* and *HygB*, three additional homozygous mutants contained tandem integrations of the selection markers without an apparent LOH at the target locus, which would not be expected to unspecifically affect mutant phenotypes. This efficiency is sufficient for rapidly testing the involvement of candidate genes in a phenotype under study.

In further experiments, we used deletion cassettes containing long flanking sequences of the target genes, assuming that this would enhance the frequency of separate integration of each marker into the target locus and allow a better comparison of the outcomes when selecting for both markers versus selection for only one resistance marker. While such deletion cassettes can be obtained by overlap PCR without a cloning step, we chose to clone the deletion cassettes in a plasmid vector to ensure that both selection markers contained identical flanking sequences and that highly pure deletion cassettes were used for transformations. The preferential integration of the *GRP2* deletion cassettes into *GRP2* allele 2 was likely due to the fact that the cloned flanking sequences were derived from this allele and contained eight mismatches in the upstream region and two mismatches in the downstream region compared to allele 1. The biased marker integration into *OYE23* allele 2 can be similarly explained.

Overall, our results show that selection for both resistance markers increased the frequency of homozygous mutants among the transformants. This being said, homozygous mutants were also obtained when selecting for only one marker, albeit with various efficiencies (between 5% and 42%; see Table 1), demonstrating (and confirming previous studies) that the use of a single selection marker is sufficient to obtain homozygous deletion mutants in a single transformation step when using Cas9 and a gene-specific gRNA. Of further note, all analyzed hygromycin-resistant transformants obtained with Cas9/gRNA contained the *HygB* marker, because the strongly increased transformation efficiency reduced the proportion of the rare spontaneously resistant clones to negligible levels.

**TABLE 1:** Frequencies of homozygous deletion mutants

Target gene	Selection	Δ/Δ frequency^*a*^	Δ/Δ with LOH
*GRP2*	Nou	8/24	3/8
	Hyg	10/24	1/10
	Nou + Hyg	24/24	10/24
*GLX3*	Nou	3/24	1/3
	Hyg	3/24	2/3
	Nou + Hyg	24/24	15/24
*OYE23*	Nou	3/24	3/3
	Hyg	3/24	1/3
	Nou + Hyg	22/24	4/22
*OYE32*	Nou	1/22	0/1
	Hyg	7/24	3/7
	Nou + Hyg	18/24	7/18

^*a*^ Not including homozygous deletion mutants with tandem integrations.

Our main motivation to simultaneously use two different selection markers for Cas9-facilitated, single-step generation of homozygous mutants was the expectation that independent integrations of *caSAT1* and *HygB* in the two alleles of a target gene would decrease the risk of an undesired LOH at the target locus, which may occur when a marker is integrated into only one target allele, and the altered chromosome then serves as a template to repair the Cas9-mediated double-strand break on the homologous chromosome. LOH, which was rarely observed in heterozygous mutants containing only one allelic replacement, was indeed a significant problem when using Cas9 for the generation of homozygous mutants. Surprisingly, this was not ameliorated by the simultaneous use of *caSAT1* and *HygB*. A possible scenario that could explain this finding is that a Cas9-mediated double-strand break in one allele of the target locus is first repaired using the homologous chromosome as a template and results in LOH before subsequent insertion of the selection markers into both chromosomes. Such LOH events can involve large regions on the same chromosome arm after break-induced replication and cause altered phenotypes that are unrelated to the deletion of the target gene. We always tested for LOH events between the target site and the telomere, which is the region that is most likely to be affected by break-induced replication, but it has been demonstrated that LOH can also extend for longer regions from the break site toward the centromere (52). Indeed, many *glx3*Δ mutants exhibited an LOH event at the centromere-proximal SpeI site, which is located 1 kb upstream of *GLX3* allele 1, in some cases without a concomitant LOH at the telomere-proximal PstI site in the downstream region (see Fig. S4; Table S2). Therefore, the observed LOH frequency is probably an underestimation of undesired LOH events on the target chromosome.

Interestingly, we also obtained mixed clones consisting of subpopulations containing either the wild-type gene, the *caSAT1* marker, or the *HygB* marker on the same chromosome. This indicates that marker integration events also occur during colony growth in some, but not other cells, especially as long as the Cas9 enzyme is still present in the cells (subpopulations may also contain aneuploid cells with chromosome duplications). This was observed because we analyzed the primary colonies appearing on the selection plates. Consequently, LOH events were also observed in subpopulations of these clones. Such mixed clones would be resolved after further restreaking for single colonies.

While the frequency of Cas9-induced LOH may depend on the transformation method (e.g., electroporation versus other protocols and use of expression cassettes for Cas9 and gRNA versus purified Cas9/gRNA complexes) and the genetic background (note that we used strains containing a hyperactive Mrr1, in which all tested target genes were constitutively overexpressed), and alterations in other genomic regions can be induced by the transformation stress, the findings of our study indicate that checking mutants for LOH at the target locus is especially important when their construction involved Cas9-mediated chromosome breakage.

## MATERIALS AND METHODS

### Strains and growth conditions

The *C. albicans* wild-type reference strain SC5314 (53) and its derivatives SCMRR1R34A and SCMRR1R34B, which contain the P683S GOF mutation in both *MRR1* alleles (*MRR1*^P683S^-*FRT*/*MRR1*^P683S^*-FRT*) (13), were used for the experiments described in this study. Strains were routinely grown in YPD liquid medium (10 g yeast extract, 20 g peptone, and 20 g glucose per liter) at 30°C in a shaking incubator. For the selection of transformants, 200 µg/mL nourseothricin (Werner Bioagents) and/or 1 mg/mL hygromycin B was added to YPD agar plates containing 15 g agar per liter.

### Plasmid constructions

To generate a *GRP2* deletion cassette, ca. 0.5 kb of *GRP2* upstream and downstream sequences was amplified by PCR with primers GRP2.13/GRP2.14 and GRP2.15/GRP2.16, respectively (all oligonucleotide primers used in this study are listed in Table S5). The PCR products were digested with ApaI/XhoI (upstream sequence) and PstI/SacII (downstream sequence), gel-purified, and ligated together with an XhoI-PstI fragment from plasmid pNIM1 (54) containing the *caSAT1* selection marker in the ApaI/SacII-digested pNIM1 to generate pGRP2M1. An XhoI-PstI fragment containing the *HygB* selection marker from pSNF1ex3 (55) was then substituted for the *caSAT1* marker to obtain pGRP2M2. Deletion cassettes containing the *caSAT1* and *HygB* selection markers for other Mrr1 target genes were generated in the same way using the gene-specific primers listed in Table S5.

### *C. albicans* transformation

Strains were transformed by electroporation (51) with the gene deletion cassettes described above. *GRP2* deletion was also performed with PCR-amplified selection markers obtained with primers GRP2.11 and GRP2.12 (Table S5), which bind to the upstream and downstream sequences of both *caSAT1* and *HygB* and contain 90 and 91 additional nucleotides corresponding to sequences at the start and end, respectively, of the *GRP2* coding sequence.

RNP complexes were generated using the CRISPR-Cas9 system from Integrated DNA Technologies, Inc., basically as reported by Grahl et al. (17). The gRNA was generated by mixing equimolar concentrations (2 µM final) of the gene-specific crRNA and the universal tracrRNA, with a final volume of 3.6 µL per transformation. This mixture was incubated at 95°C for 5 minutes and allowed to cool down at room temperature. To assemble the RNP complex, 3.6 µL of the gRNA was mixed with 3 µL of 4 µM Alt-R S.p. Cas9 nuclease V3 and incubated at room temperature for 5 minutes. For each transformation, 40 µL of competent cells was mixed with 6.6 µL of the RNP complex plus 100 ng of each repair template and transferred to an electroporation cuvette (0.2 cm). Electroporation was performed with a 1,800-V pulse. After electroporation, the cells were resuspended in 1 mL YPD and incubated at 30°C for 4 h with shaking. Appropriate dilutions of the recovered cells were spread on YPD plates supplemented with 200 µg/mL nourseothricin, 1 mg/mL hygromycin B, or both antibiotics. After 2 days of incubation at 30°C, single colonies were picked for further analysis.

### Genetic analysis of mutants

Genomic DNA from *C. albicans* strains was isolated as described previously (51). The DNA was digested with appropriate restriction enzymes, separated on an agarose gel (0.8%–1.0%), transferred by vacuum blotting onto a nylon membrane, and fixed by UV crosslinking. Southern hybridization with enhanced chemiluminescence-labeled probes (including a labeled size marker) was performed with the Amersham ECL Direct Nucleic Acid Labelling and Detection System (Cytiva) according to the instructions of the manufacturer. The upstream and downstream sequences of the deletion cassettes were used as gene-specific probes. An additional probe specific for the *OYE32* coding sequence (to distinguish the band corresponding to *OYE32-1* from that corresponding to *oye32-2*∆::*HygB*, see Fig. S6A and D) was amplified with primers OYE32.05/OYE32.06. Marker-specific probes were amplified with primers SAT1.01/SAT1.04 and HygB-3/HygB-7, respectively, thereby excluding sequences that are contained in both *caSAT1* and *HygB*. The sequences of tandem integrations of the selection markers with short homology regions in the *GRP2* locus were determined by amplifying the fusion between the *HygB* and *caSAT1* markers with primers HygB-5 and SAT8, followed by sequencing. The order of the markers in clone B2 was determined by PCR with the primer pairs HygB-5/GRP2.16 and SAT1.03/GRP2.16.

### Fluconazole minimal inhibitory concentration assays

The fluconazole susceptibilities of the strains were determined by a previously described broth microdilution method (56), with slight modifications. A 2-day-old colony from a YPD agar plate was suspended in 2 mL of a 0.9% NaCl solution, and 4 µL of the suspension was mixed with 2 mL 2× SD-CSM medium [13.4 g yeast nitrogen base without amino acids (MP Biomedicals), 40 g glucose, and 1.58 g complete supplement medium (MP Biomedicals) per liter]. A twofold dilution series of fluconazole (Sigma) was prepared in water, starting from an initial concentration of 512 µg/mL. One hundred microliters of each fluconazole solution was then mixed with 100 µL of the cell suspension in a 96-well microtiter plate, and the plates were incubated for 24 h at 37°C. The MIC of fluconazole was defined as the drug concentration that abolished or drastically reduced visible growth compared to a drug-free control.

## SUPPLEMENTAL MATERIAL

Fig. S1 msphere.00388-24-s0001.docx *HygB-caSAT1* fusion sequences.

Fig. S2 msphere.00388-24-s0002.pdf Recovery of transformants after electroporation with the *GRP2* deletion cassettes.

Fig. S3 msphere.00388-24-s0003.pdf Deletion of *GRP2* using the *caSAT1* and *HygB* selection markers with long flanking sequences.

Fig. S4 msphere.00388-24-s0004.pdf Deletion of *GLX3* using the *caSAT1* and *HygB* selection markers.

Fig. S5 msphere.00388-24-s0005.pdf Deletion of *OYE23* using the *caSAT1* and *HygB* selection markers.

Fig. S6 msphere.00388-24-s0006.pdf Deletion of *OYE32* using the *caSAT1* and *HygB* selection markers.

Supplemental tables msphere.00388-24-s0007.xlsx Tables S1 to S6.

ASM does not own the copyrights to Supplemental Material that may be linked to, or accessed through, an article. The authors have granted ASM a non-exclusive, world-wide license to publish the Supplemental Material files. Please contact the corresponding author directly for reuse.
