# Supplementary material for: Probing gene function in Candida albicans wild-type strains by Cas9-facilitated one-step integration of two dominant selection markers: a systematic analysis of recombination events at the target locus
Source: mSphere. 2024 Jun 28;9(7):e00388-24. doi: 10.1128/msphere.00388-24 (PMC11288041; doi:10.1128/msphere.00388-24)
Supplement: Fig. S1 — HygB-caSAT1 fusion sequences. [file msphere.00388-24-s0001.docx]

***HygB-caSAT1* fusion sequences**

…AATAAATAATTAGATAAGGGTGGTAATTATTACTATTTACAATCAAAGGTGGTCCTGCAG (End *HygB*)

ACGATTCGGTTGCTCAAATTATTGGTTAAATGAATGATATTCAGATTGAATGATGATTAGATTGAACTTTTTTTTACTCCTTAGATACAAT (3‘*GRP2*)

TTACAATTTTCTTATAATCAATTAAAAAATATCTTCATATAACTACTATAATGTCTTCATCTACTACAGTTTTCGTTTCTGGTGCTTCTG (5‘*GRP2*)

CTCGAGCGTCAAAACTAGAGAATAATAAAGAAAACGATCTTTTCAAAAAGAAAAAACCTT… (Start *caSAT1*)

Red: End of *HygB* marker

Blue: Start of *caSAT1* marker

Yellow: Restriction sites (PstI and XhoI) flanking the markers

Light green: *GRP2* downstream short homology region contained in the deletion cassettes (stop codon underlined)

Dark green: *GRP2* upstream short homology region contained in the deletion cassettes (start codon underlined)

Clone A1

TAACATATGTATTATGTGAAGTGTGAAGGGGGAGATTTTCACTTTATTAGATTTGTATATATGTATAATAAATAAATAAATAAGTTAAATAAATAATTAGATAAGGGTGGTAATTATTACTATTTACAATCAAAGGTGGTCCTGCAGACGATTACAATTTTCTTATAATCAATTAAAAAATATCTTCATATAACTACTATAATGTCTTCATCTACTACAGTTTTCGTTTCTGGTGCTTCTGCTCGAGCGTCAAAACTAGAGAATAATAAAGAAAACGATCTTTTCAAAAAGAAAAAACCTTTTAGTTTTCCTTTGTTGTTGTTGTGGGTGTGTGCTATTTATATTATATAGTTTACTCATAATACCATAAAATATTCGGTTTGATTAGGTTATTTTAATAAGCTAATTTGTTTCTAATCGTGTAATTTATGCTGTGTATATTAAGTAGTGTGTGCACTGCCCAAAAATGTTTGTTGTTTATAGTCGGTTAAAGAGAAAAAAGAAAAAAAGATCCATACACACACGTTAATTAGTTGTTCAACGTAATACACTCATATTTTGTTCTTATTTGCTTTCGGTCGCTGTTCTCACCAAGATTTATTGCCAACGAAACAATTTTTTTTTATATATTTTCAGATTTTTCTTTTTTTCCTTTCCTTTCCTTTTCTAATTTTCACTCCTGGTTTTCTTTCTTTCTTAGAAACATTATCTCGATATTAATATTAAAAAAATATAATCATTCAAATG

Clone A4

TAACATATGTATTATGTGAAGTGTGAAGGGGGAGATTTTCACTTTATTAGATTTGTATATATGTATAATAAATAAATAAATAAGTTAAATAAATAATTAGATAAGGGTGGTAATTATTACTATTTACAATCAAAGGTGGTCCTGCAGACGATTCGGTTGCTCAAATTATTGGTTAAATGAATGATATTCAGATTGAATGATGATTAGATTGAACTTTTTTTTACTCCTTAGATACAATTTTCTTATAATCAATTAAAAAATATCTTCATATAACTACTATAATGTCTTCATCTACTACAGTTTTCGTTTCTGGTGCTTCTGCTCGAGCGTCAAAACTAGAGAATAATAAAGAAAACGATCTTTTCAAAAAGAAAAAACCTTTTAGTTTTCCTTTGTTGTTGTTGTGGGTGTGTGCTATTTATATTATATAGTTTACTCATAATACCATAAAATATTCGGTTTGATTAGGTTATTTTAATAAGCTAATTTGTTTCTAATCGTGTAATTTATGCTGTGTATATTAAGTAGTGTGTGCACTGCCCAAAAATGTTTGTTGTTTATAGTCGGTTAAAGAGAAAAAAGAAAAAAAGATCCATACACACACGTTAATTAGTTGTTCAACGTAATACACTCATATTTTGTTCTTATTTGCTTTCGGTCGCTGTTCTCACCAAGATTTATTGCCAACGAAACAATTTTTTTTTATATATTTTCAGATTTTTCTTTTTTCCTTTCCTTTCCTTTTCTAATTTTCACTCCTGGTTTTCTTTCTTTCTTAGAAACATTATCTCGATATTAATATTAAAAAATATAATCATTCAAATG

Clone A5

TAACATATGTATTATGTGAAGTGTGAAGGGGGAGATTTTCACTTTATTAGATTTGTATATATGTATAATAAATAAATAAATAAGTTAAATAAATAATTAGATAAGGGTGGTTACAATTTTCTTATAATCAATTAAAAAATATCTTCATATAACTACTATAATGTCTTCATCTACTACAGTTTTCGTTTCTGGTGCTTCTGCTCGAGCGTCAAAACTAGAGAATAATAAAGAAAACGATCTTTTCAAAAAGAAAAAACCTTTTAGTTTTCCTTTGTTGTTGTTGTGGGTGTGTGCTATTTATATTATATAGTTTACTCATAATACCATAAAATATTCGGTTTGATTAGGTTATTTTAATAAGCTAATTTGTTTCTAATCGTGTAATTTATGCTGTGTATATTAAGTAGTGTGTGCACTGCCCAAAAATGTTTGTTGTTTATAGTCGGTTAAAGAGAAAAAAGAAAAAAAGATCCATACACACACGTTAATTAGTTGTTCAACGTAATACACTCATATTTTGTTCTTATTTGCTTTCGGTCGCTGTTCTCACCAAGATTTATTGCCAACGAAACAATTTTTTTTTATATATTTTCAAATTTTTCTTTTTTTCCTTTCCTTTCCTTTTCTAATTTTCACTCCTGGTTTTCTTTCTTTCTTAGAAACATTATCTCGATATTAATATTAAAAAAATATAATCATTCAAAATG

Clone B2

TAACATATGTATTATGTGAAGTGTGAAGGGGGAGATTTTCACTTTATTAGATTTGTATATATGTATAATAAATAAATAAATAAGTTAAATAAATAATTAGATAAGGGTGGTAATTATTACTATTTACAATCAAAGGTGGTCCTGCAGACGATTCGGTTGCTCAAATTATTGGTTAAATGAATGATATTCAGATTGAATTTACAATTTTCTTATAATCAATTAAAAAATATCTTCATATAACTACTATAATGTCTTCATCTACTACAGTTTTCGTTTCTGGTGCTTCTGCTCGAGCGTCAAAACTAGAGAATAATAAAGAAAACGATCTTTTCAAAAAGAAAAAACCTTTTAGTTTTCCTTTGTTGTTGTTGTGGGTGTGTGCTATTTATATTATATAGTTTACTCATAATACCATAAAATATTCGGTTTGATTAGGTTATTTTAATAAGCTAATTTGTTTCTAATCGTGTAATTTATGCTGTGTATATTAAGTAGTGTGTGCACTGCCCAAAAATGTTTGTTGTTTATAGTCGGTTAAAGAGAAAAAAGAAAAAAAGATCCATACACACACGTTAATTAGTTGTTCAACGTAATACACTCATATTTTGTTCTTATTTGCTTTCGGTCGCTGTTCTCACCAAGATTTATTGCCAACGAAACAATTTTTTTTTATATATTTTCAGATTTTTCTTTTTTCCTTTCCTTTCCTTTTCTAATTTTCACTCCTGGTTTTCTTTCTTTCTTAGAAACATTATCTCGATATTAATATTAAAAAAATATAATCATTCAAATG

Clone B6

TAACATATGTATTATGTGAAGTGTGAAGGGGGAGATTTTCACTTTATTAGATTTGTATATATGTATAATAAATAAATAAATAAGTTAAATAAATAATTAGATAAGGGTGGTAATTATTACTATTTACAATCAAAGGTGGTCCTGCAGACGATTCGGTTGCTCAAATTATTGGTTAAATGAATGATATTCAGATTGAATGATGATTAGATTGAACTTTTTTTTACTCCTTAGATACAATGTTTGTTGTTTATAGTCGGTTAAAGAGAAAAAAGAAAAAAAGATCCATACACACACGTTAATTAGTTGTTCAACGTAATACACTCATATTTTGTTCTTATTTGCTTTCGGTCGCTGTTCTCACCAAGATTTATTGCCAACGAAACAATTTTTTTTTATATATTTTCAGATTTTTCTTTTTTTCCTTTCCTTTCCTTTTCTAATTTTCACTCCTGGTTTTCTTTCTTTCTTAGAAACATTATCTCGATATTAATATTAAAAAAATATAATCATTCAAAATG

**FIG S1** *HygB-caSAT1* fusion sequences.
