# Supplementary material for: Probing gene function in Candida albicans wild-type strains by Cas9-facilitated one-step integration of two dominant selection markers: a systematic analysis of recombination events at the target locus
Source: mSphere. 2024 Jun 28;9(7):e00388-24. doi: 10.1128/msphere.00388-24 (PMC11288041; doi:10.1128/msphere.00388-24)
Supplement: Fig. S2 — Recovery of transformants after electroporation with the GRP2 deletion cassettes. [file msphere.00388-24-s0002.pdf]

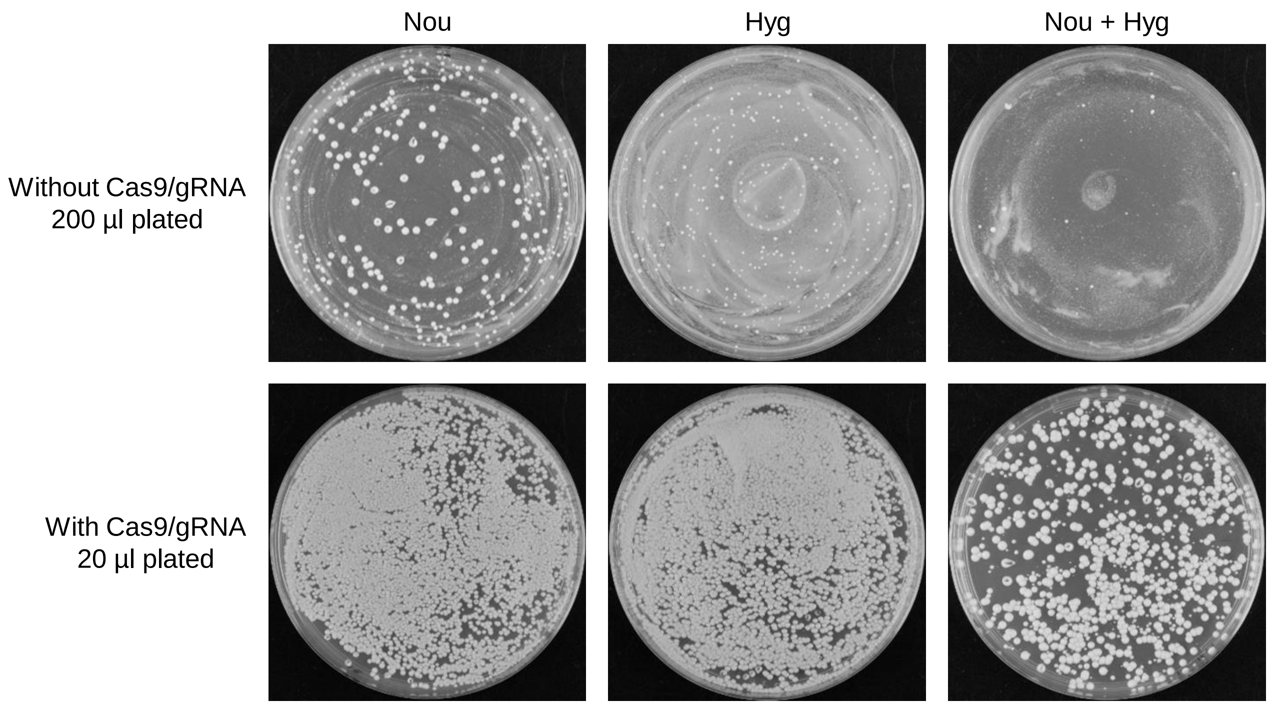

FIG S2 Recovery of transformants after electroporation with the *GRP2* deletion cassettes in the absence of Cas9/gRNA (top panels, 200 µl of the original cell suspension [1 ml] per plate) and in the presence of Cas9 and *GRP2*-specific gRNA (middle panels, 200 µl of a 10-fold dilution of the original cell suspension per plate). Transformants were selected on plates with 200 µg/ml nourseothrin (Nou), 1 mg/ml hygromycin (Hyg), or both antibiotics. Plates were incubated for 2 days at 30°C.
