# Supplementary material for: Probing gene function in Candida albicans wild-type strains by Cas9-facilitated one-step integration of two dominant selection markers: a systematic analysis of recombination events at the target locus
Source: mSphere. 2024 Jun 28;9(7):e00388-24. doi: 10.1128/msphere.00388-24 (PMC11288041; doi:10.1128/msphere.00388-24)
Supplement: Fig. S3 — Deletion of GRP2 using the caSAT1 and HygB selection markers with long flanking sequences. [file msphere.00388-24-s0003.pdf]

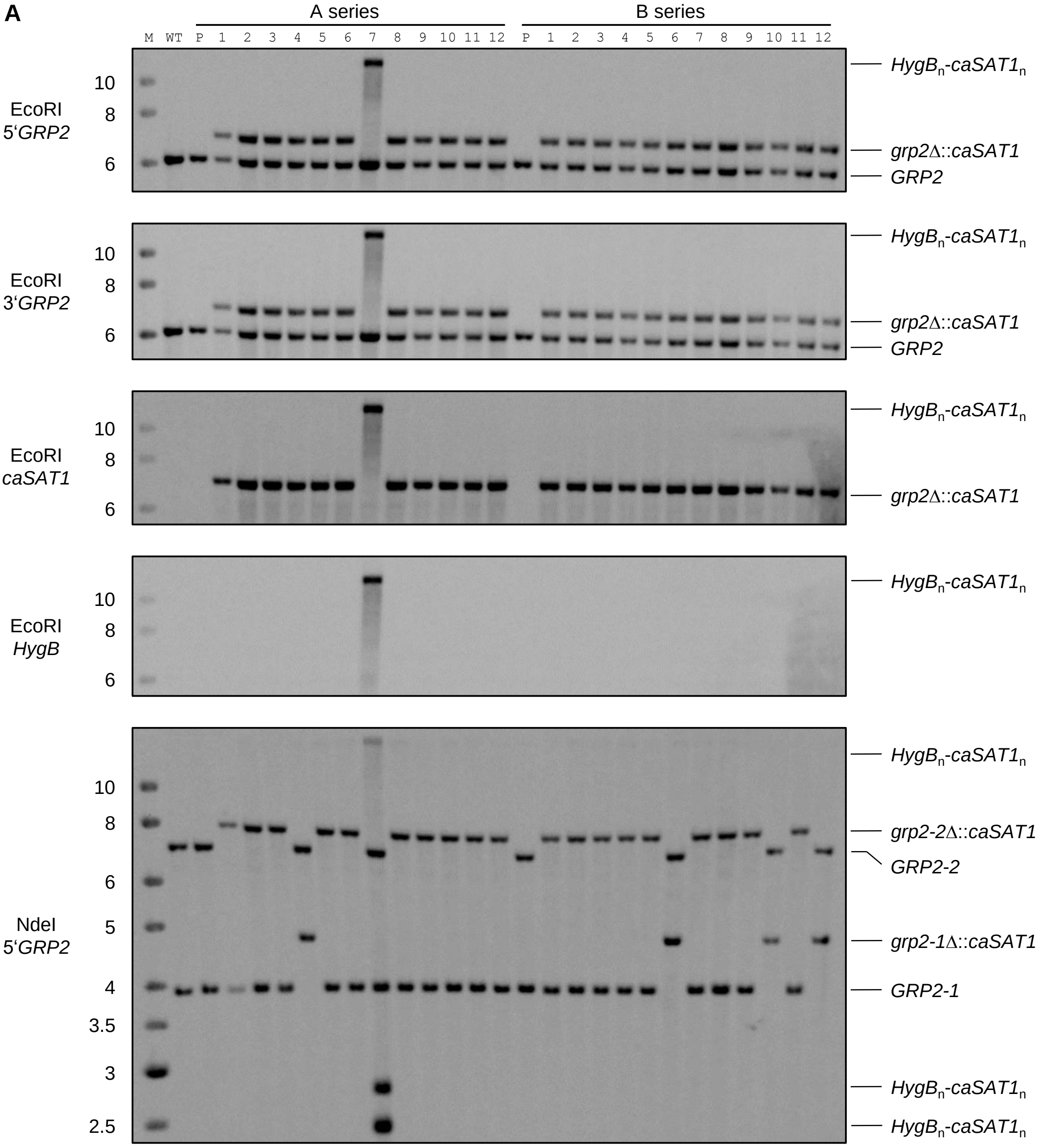

FIG S3 Deletion of *GRP2* using the *caSAT1* and *HygB* selection markers with long flanking sequences. The figure shows Southern hybridizations of EcoRI- or NdeI-digested genomic DNA of the wild-type strain SC5314 (WT), the parental strains SCMRR1R34A and SCMRR1R34B (P), and the two series of transformants derived from them with the probes specified on the left. The upstream and downstream EcoRI sites (see Fig.1A) are located outside of the cloned *GRP2* flanking sequences. The identities of the hybridizing fragments are indicated on the right side of the blots. M, size markers (in kb). (A, this page): Clones A1-A12 and B1-B12 obtained after transformation without Cas9/gRNA and selection on nourseothricin plates. Following pages: (B) Clones A1-A12 and B1-B12 obtained after transformation without Cas9/gRNA and selection on hygromycin plates. (C) Clones A1-A13 and B1-B11 obtained after transformation without Cas9/gRNA and selection on nourseothricin + hygromycin plates. (D): Clones A1-A12 and B1-B12 obtained after transformation with Cas9/gRNA and selection on nourseothricin plates. (E) Clones A1-A12 and B1-B12 obtained after transformation with Cas9/gRNA and selection on hygromycin plates. (F) Clones A1-A12 and B1-B12 obtained after transformation with Cas9/gRNA and selection on nourseothricin + hygromycin plates.

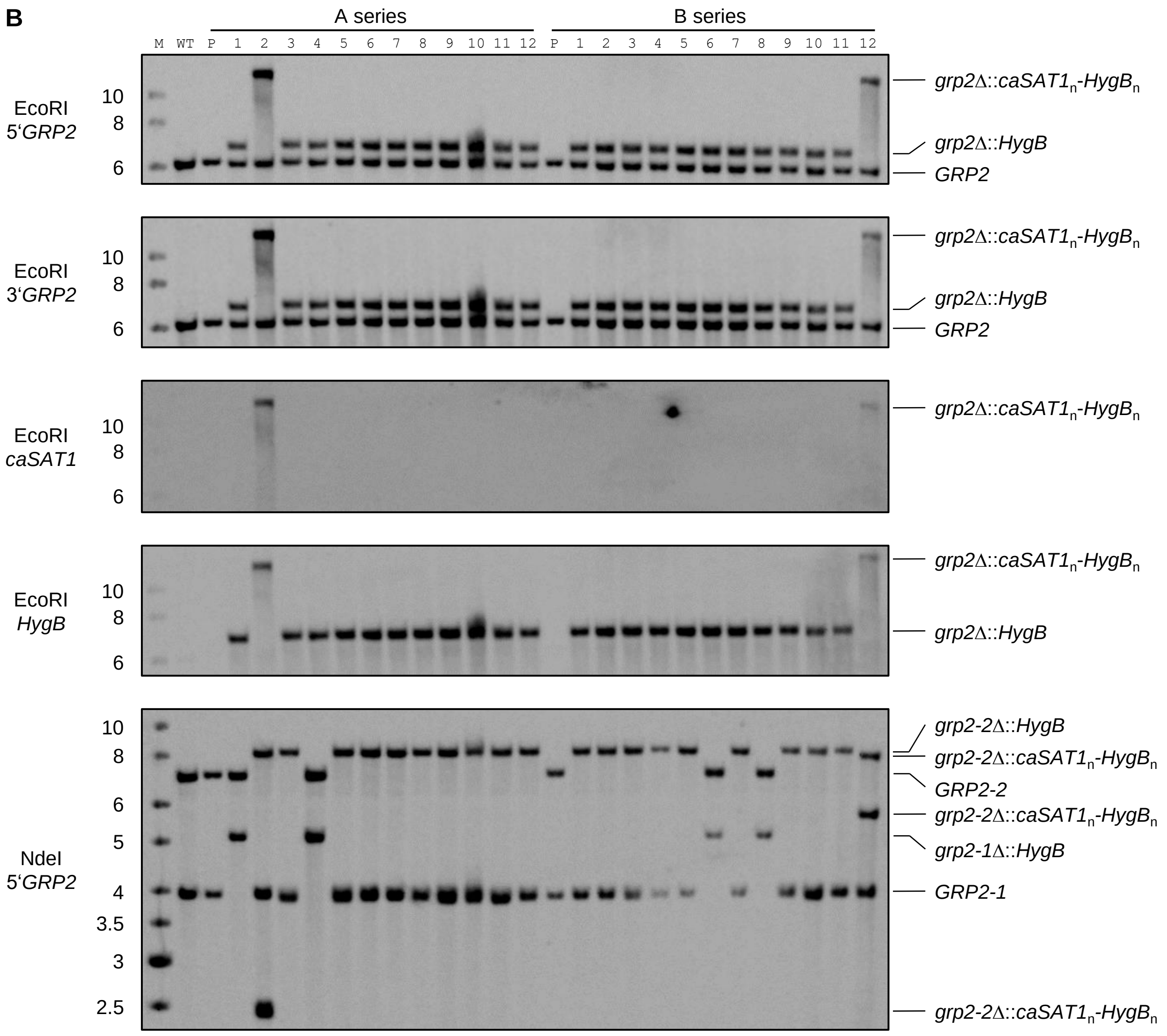

FIG S3 (continued) Clones A1-A12 and B1-B12 obtained after transformation without Cas9/gRNA and selection on hygromycin plates.

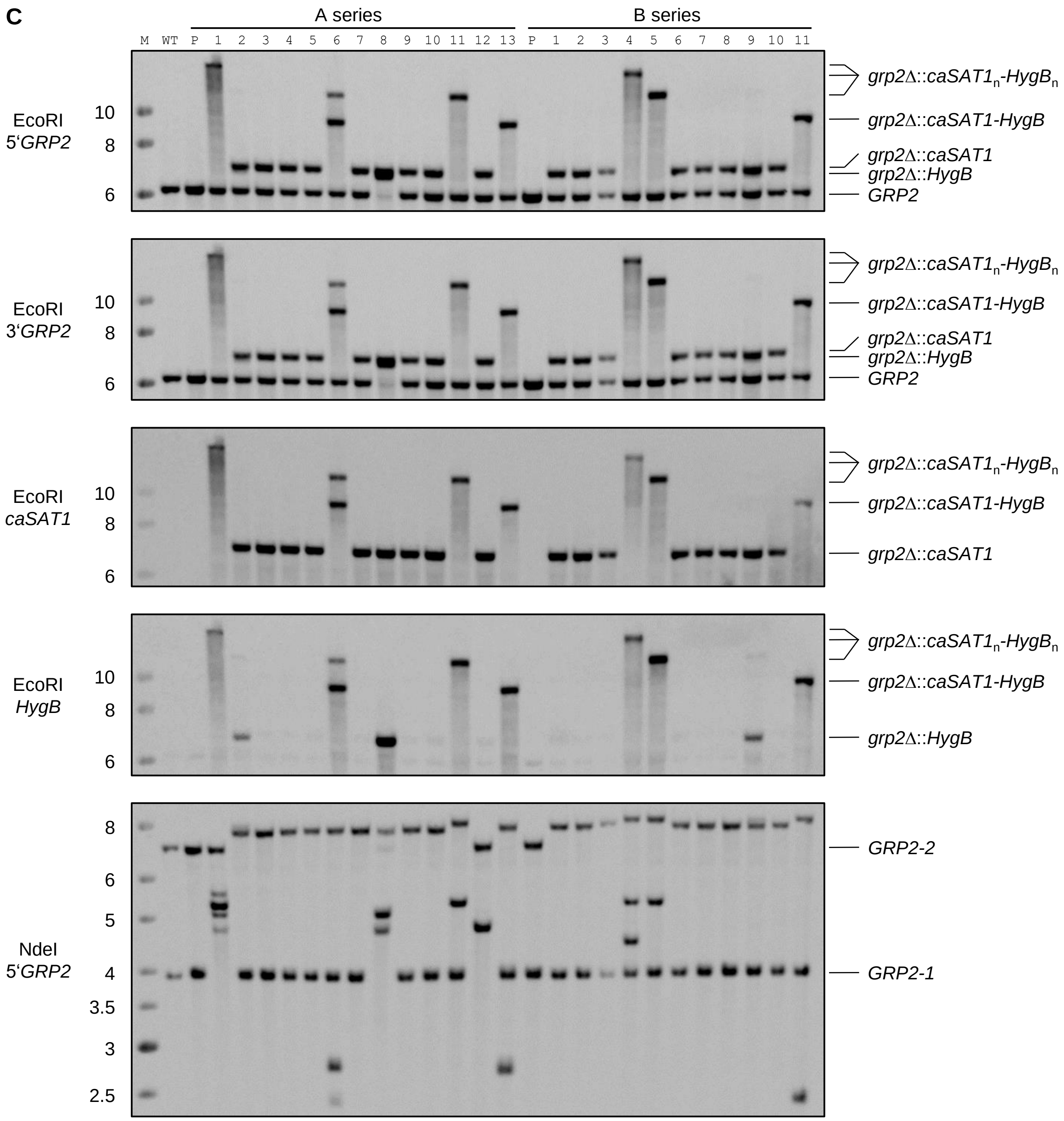

FIG S3 (continued) Clones A1-A13 and B1-B11 obtained after transformation without Cas9/gRNA and selection on nourseothricin + hygromycin plates. Unlabeld bands of the NdeI-digested DNA correspond to different integration events and are explained in Table S1.

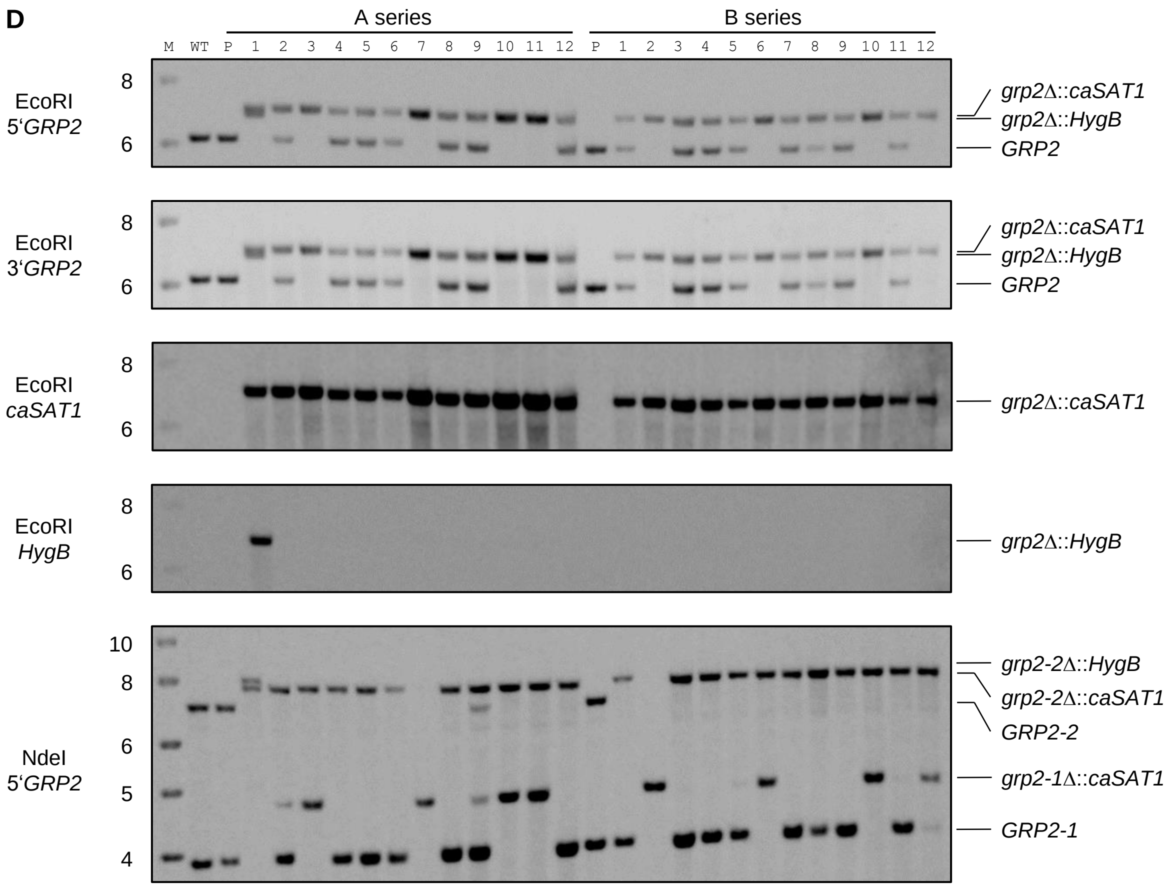

FIG S3 (continued) Clones A1-A12 and B1-B12 obtained after transformation with Cas9/gRNA and selection on nourseothricin plates.

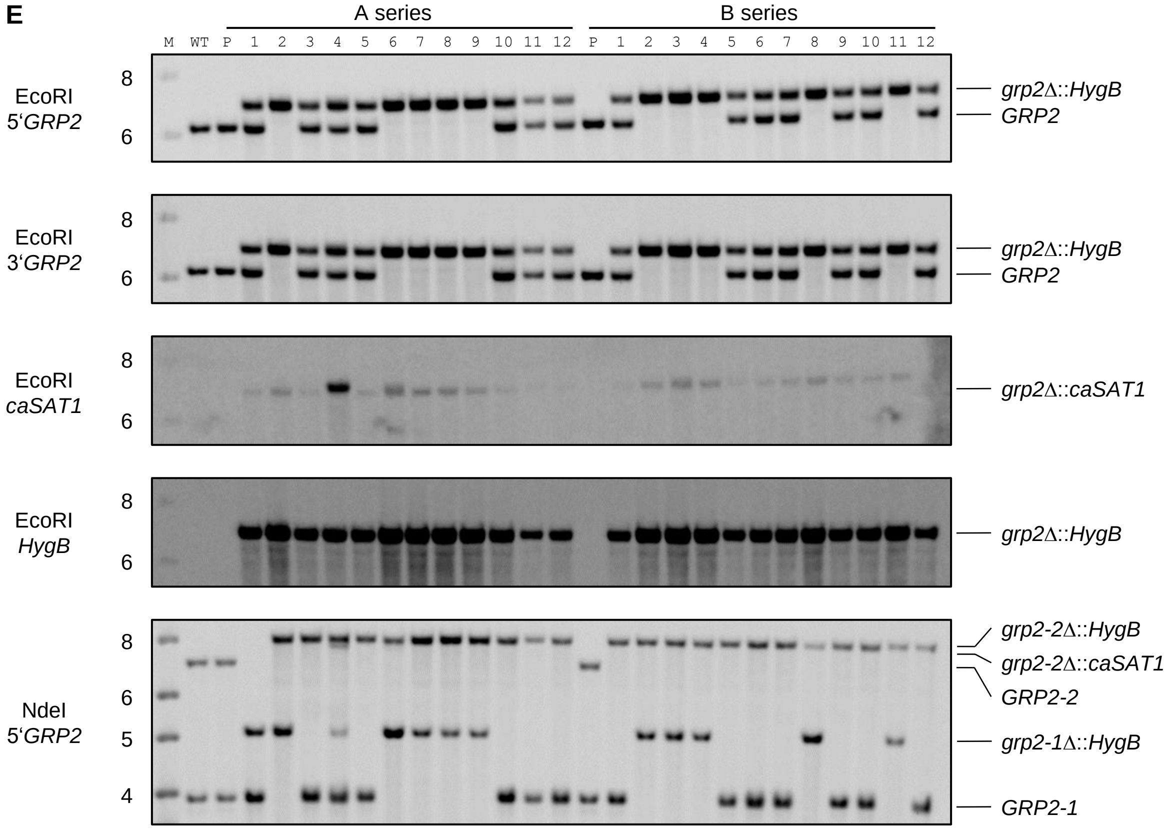

FIG S3 (continued) Clones A1-A12 and B1-B12 obtained after transformation with Cas9/gRNA and selection on hygromycin plates. The weaker bands seen in all clones after hybridization with the *caSAT1* probe are due to remaining signals from the previous hybridization with the *HygB* probe

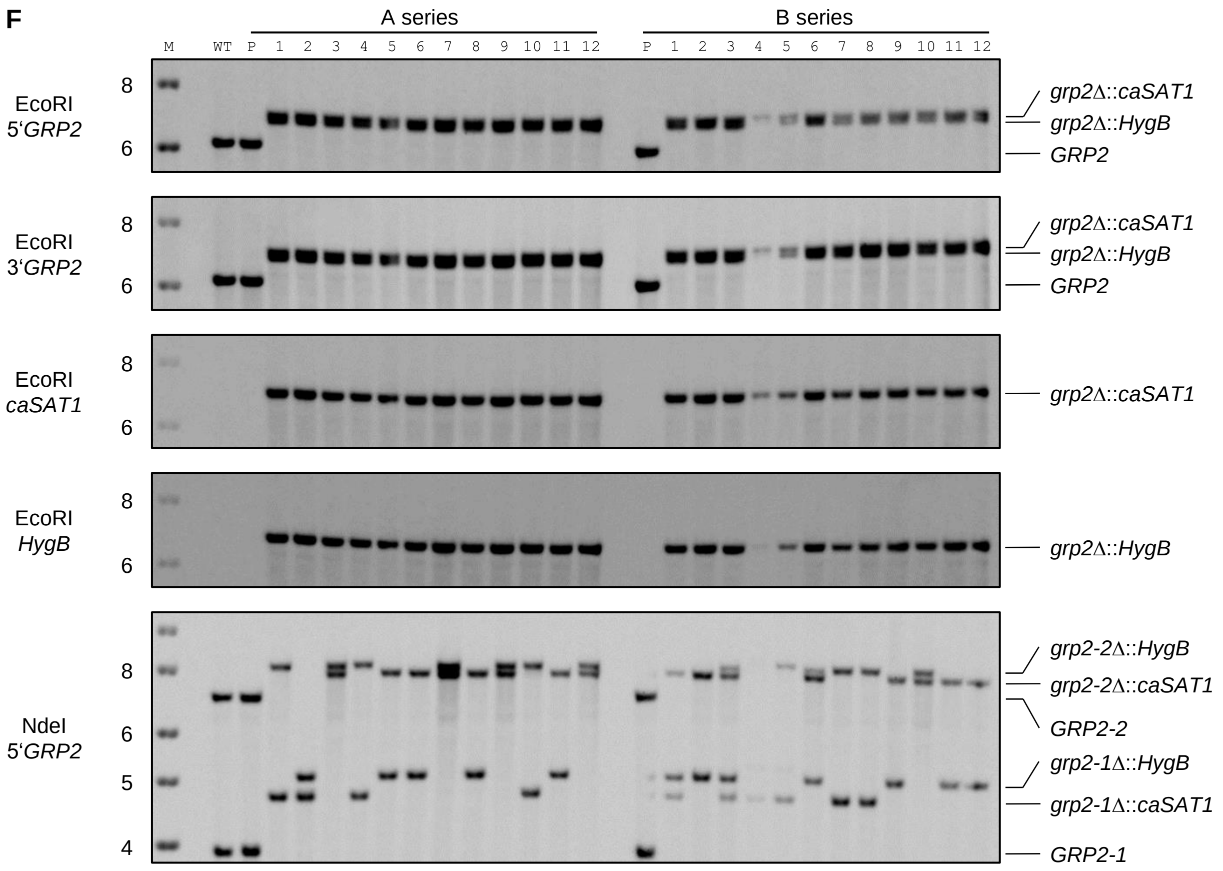

FIG S3 (continued) Clones A1-A12 and B1-B12 obtained after transformation with Cas9/gRNA and selection on nourseothricin + hygromycin plates.
