# Supplementary material for: Probing gene function in Candida albicans wild-type strains by Cas9-facilitated one-step integration of two dominant selection markers: a systematic analysis of recombination events at the target locus
Source: mSphere. 2024 Jun 28;9(7):e00388-24. doi: 10.1128/msphere.00388-24 (PMC11288041; doi:10.1128/msphere.00388-24)
Supplement: Fig. S4 — Deletion of GLX3 using the caSAT1 and HygB selection markers. [file msphere.00388-24-s0004.pdf]

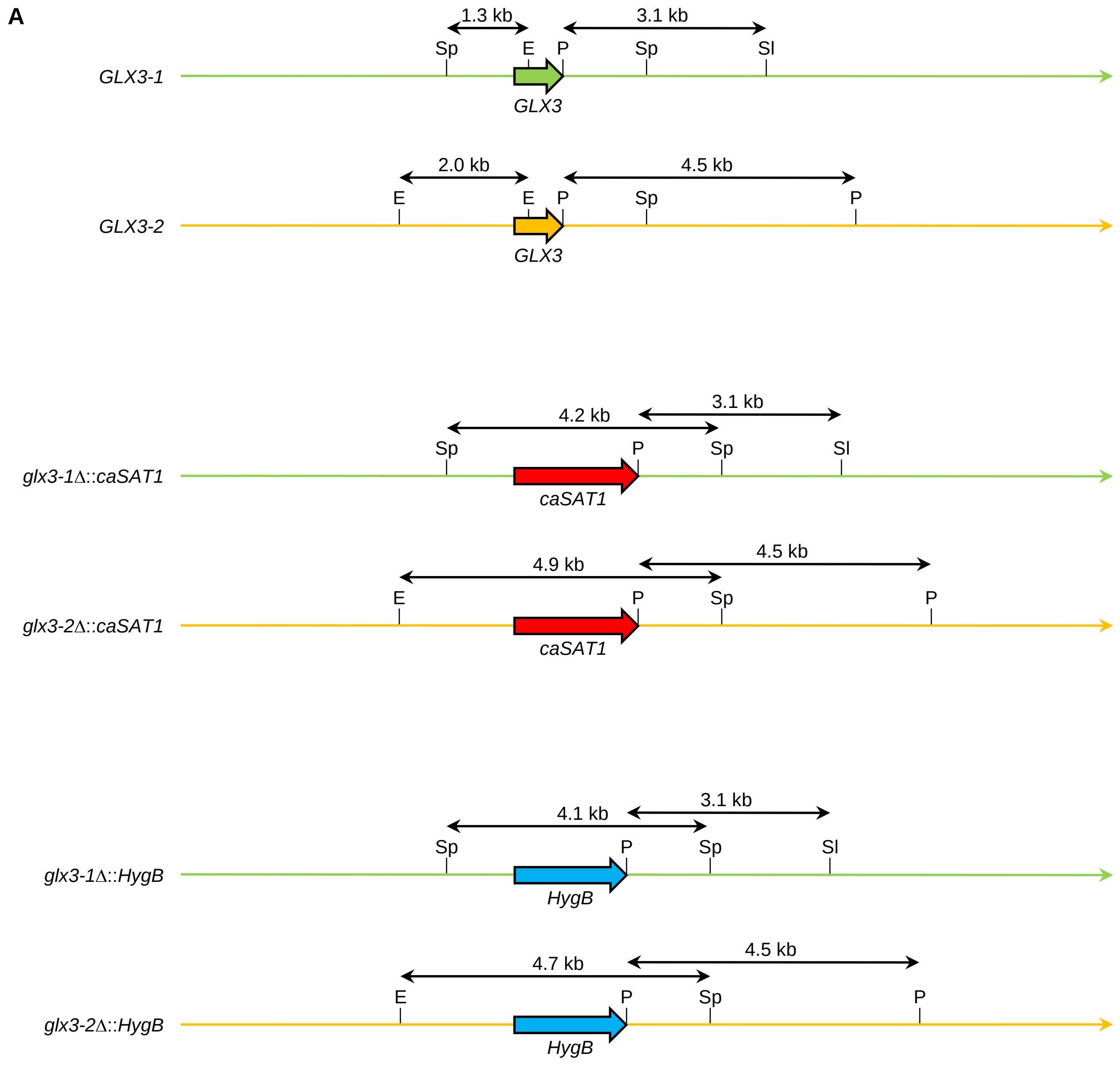

FIG S4 Deletion of *GLX3* using the *caSAT1* and *HygB* selection markers. (A) Structure of the *GLX3* locus in the wild type and in mutants containing the *caSAT1* and *HygB* selection markers in either of the two *GLX3* alleles. Arrows on the lines representing the chromosomes point towards the telomere. The locations of relevant SpeI [Sp], EcoRI [E], PstI [P], and SalI [Sl] sites and the sizes of corresponding fragments are shown. The PstI site at the end of the *GLX3* coding sequence is retained in the deletion cassettes, such that the sizes of the PstI-SalI (allele 1) and PstI-PstI (allele 2) fragments remain unchanged after insertion of the *caSAT1* and *HygB* markers. Continued on next page.

B

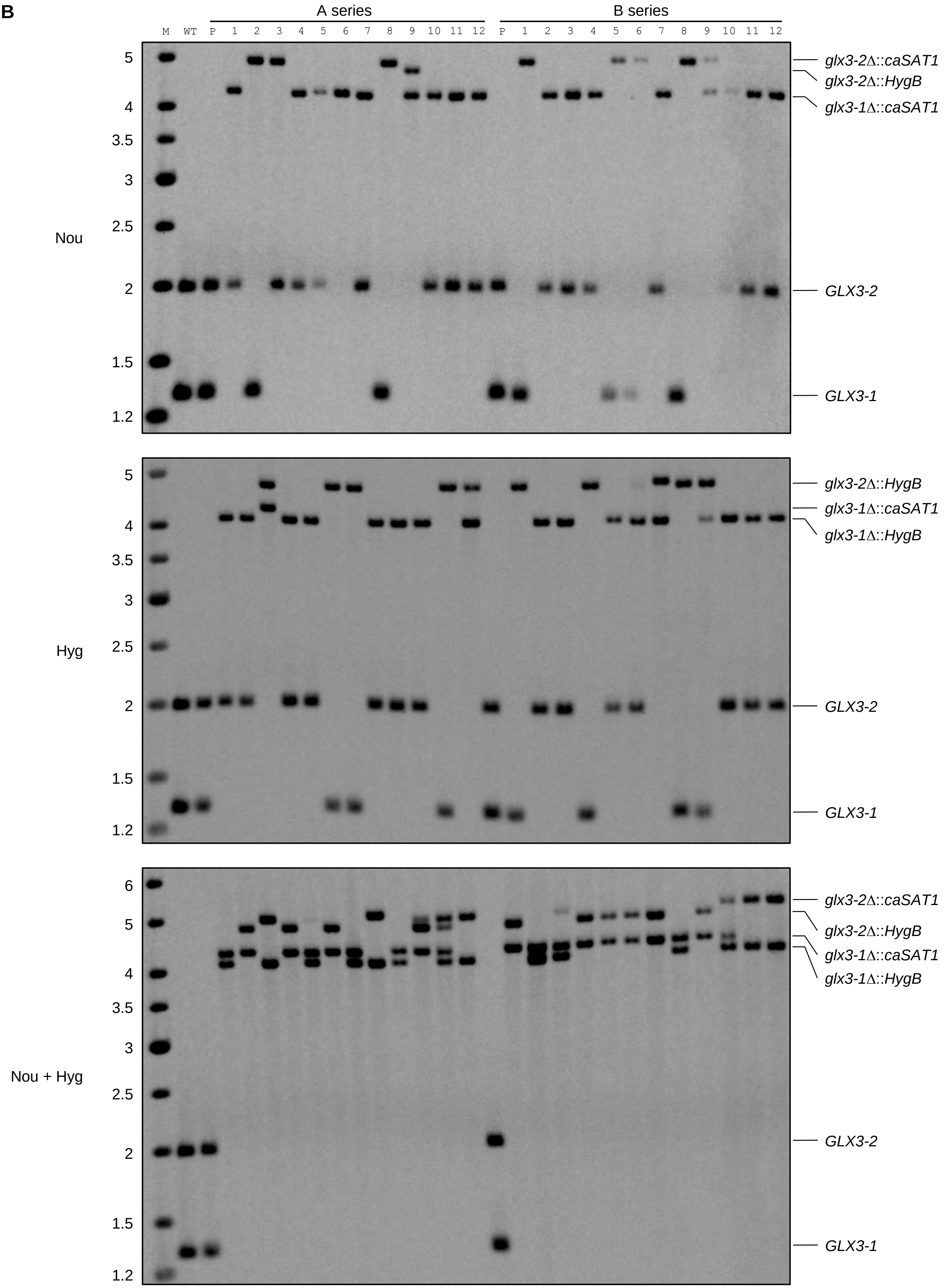

FIG S4 continued (B) Southern hybridizations of EcoRI/SpeI-digested genomic DNA of the wild-type strain SC5314 (WT), the parental strains SCMRR1R34A and SCMRR1R34B (P), and the two series of transformants derived from them after selection on plates containing nourseothricin (Nou), hygromycin (Hyg), or both nourseothricin and hygromycin (Nou + Hyg) with a 5*'GLX3* probe. The identities of the hybridizing fragments are indicated on the right side of the blots. M, size markers (in kb).

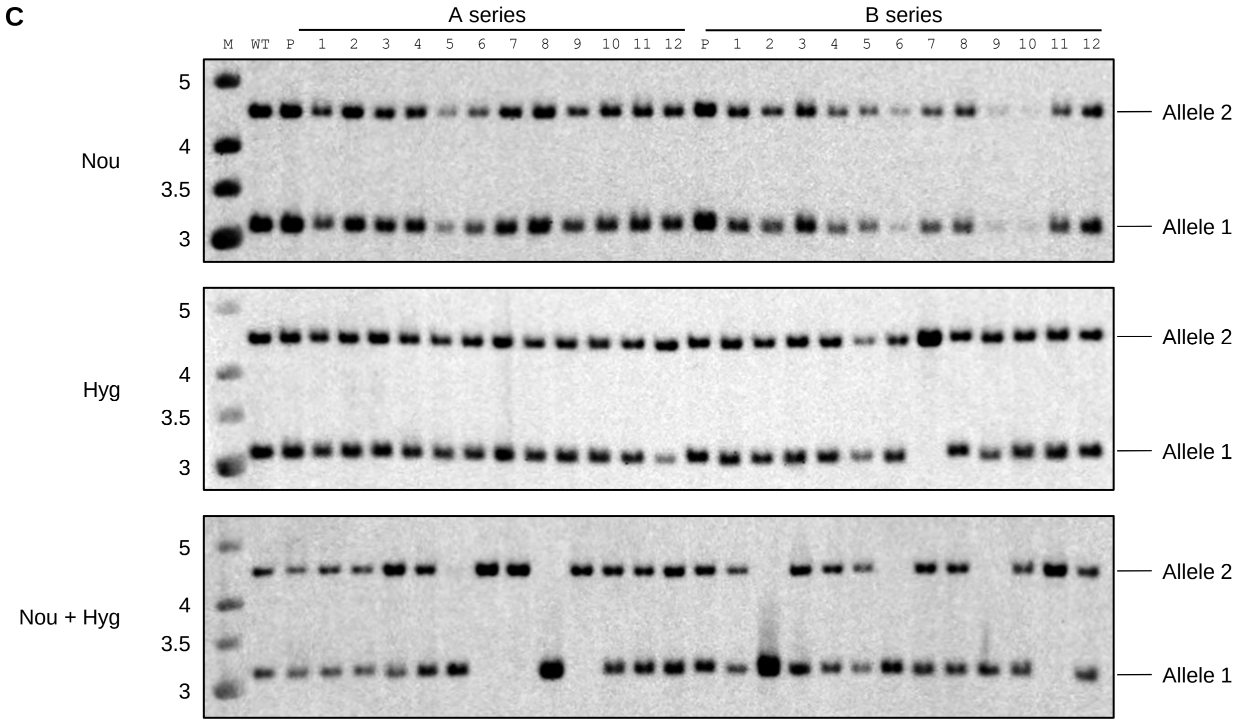

FIG S4 continued (C) Southern hybridizations of PstI/SalI-digested genomic DNA of the strains shown in (B) with a 3*'GLX3* probe. The identities of the hybridizing fragments are indicated on the right side of the blots. M, size markers (in kb).
