# Supplementary material for: Probing gene function in Candida albicans wild-type strains by Cas9-facilitated one-step integration of two dominant selection markers: a systematic analysis of recombination events at the target locus
Source: mSphere. 2024 Jun 28;9(7):e00388-24. doi: 10.1128/msphere.00388-24 (PMC11288041; doi:10.1128/msphere.00388-24)
Supplement: Fig. S5 — Deletion of OYE23 using the caSAT1 and HygB selection markers. [file msphere.00388-24-s0005.pdf]

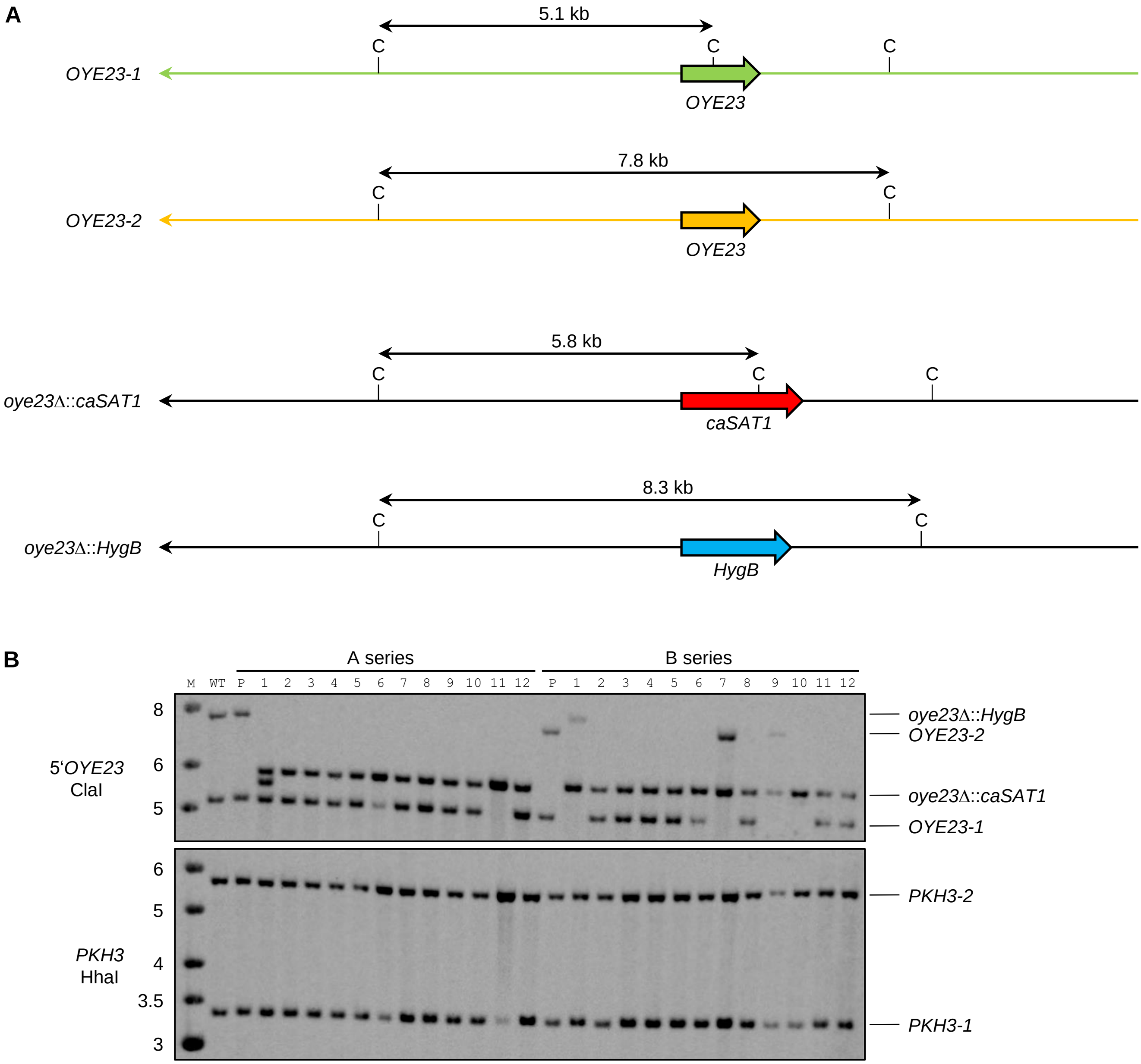

FIG S5 Deletion of *OYE23* using the *caSAT1* and *HygB* selection markers. (A) Structure of the *OYE23* locus in the wild type and in mutants containing the *caSAT1* and *HygB* selection markers in either of the two *OYE23* alleles. Arrows on the lines representing the chromosomes point towards the telomere. The locations of diagnostic ClaI [C] sites and the sizes of corresponding fragments are shown. (B) Southern hybridizations of ClaI- or HhaI-digested genomic DNA of the wild-type strain SC5314 (WT), the parental strains SCMRR1R34A and SCMRR1R34B (P), and the two series of transformants derived from them after selection on plates containing nourseothricin with a 5`*OYE23* probe and a *PKH3*-specific probe to detect LOH on the same chromosome arm. The identities of the hybridizing fragments are indicated on the right side of the blots. M, size markers (in kb). Continued on next page.

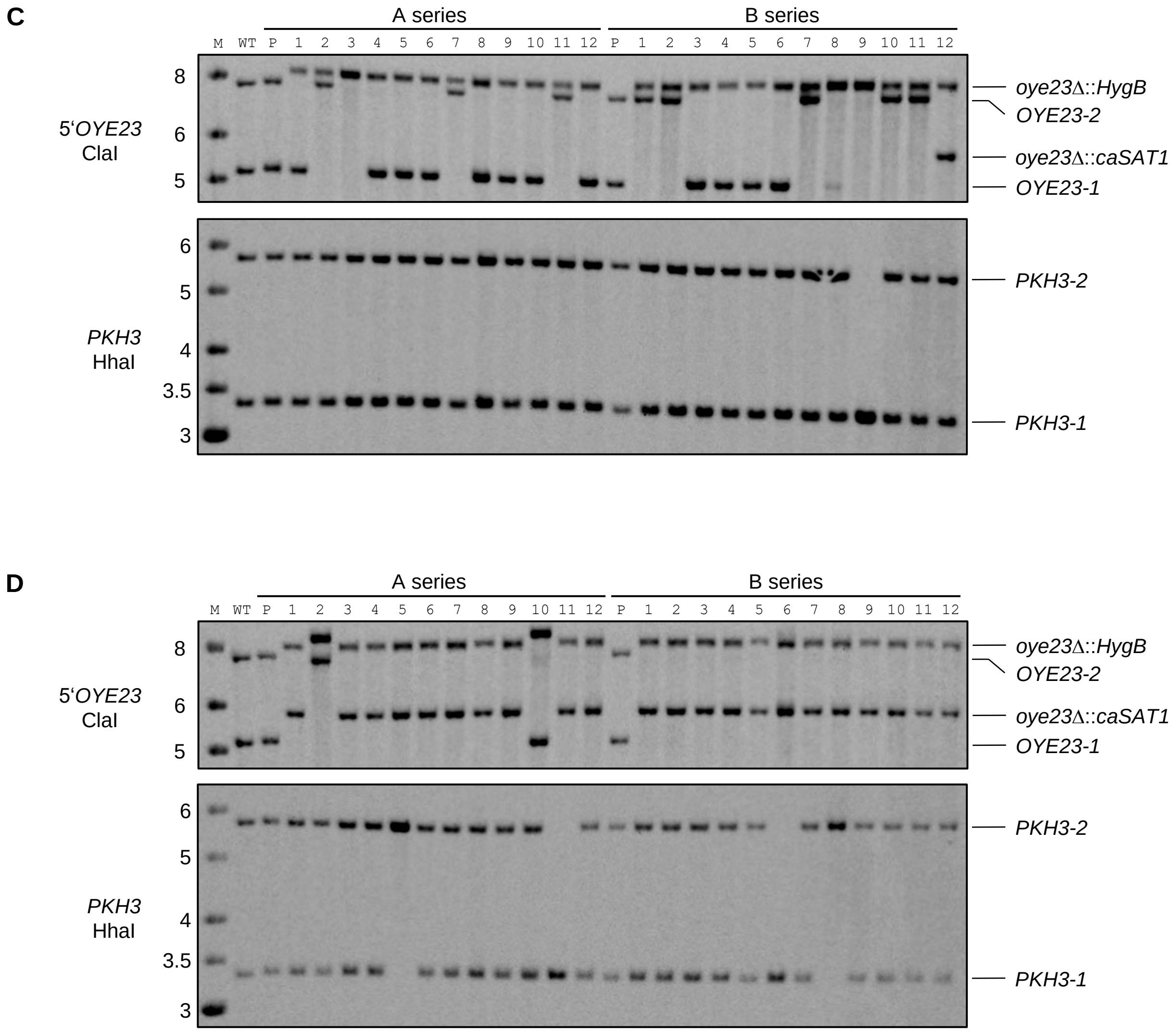

FIG S5 continued Southern hybridizations of ClaI- or HhaI-digested genomic DNA of the wild-type strain SC5314 (WT), the parental strains SCMRR1R34A and SCMRR1R34B (P), and the two series of transformants derived from them after selection on plates containing hygromycin (C) or both nourseothricin and hygromycin (D) with 5`*OYE23* and *PKH3*-specific probes. The identities of the hybridizing fragments are indicated on the right side of the blots. M, size markers (in kb).
