# Supplementary material for: Probing gene function in Candida albicans wild-type strains by Cas9-facilitated one-step integration of two dominant selection markers: a systematic analysis of recombination events at the target locus
Source: mSphere. 2024 Jun 28;9(7):e00388-24. doi: 10.1128/msphere.00388-24 (PMC11288041; doi:10.1128/msphere.00388-24)
Supplement: Fig. S6 — Deletion of OYE32 using the caSAT1 and HygB selection markers. [file msphere.00388-24-s0006.pdf]

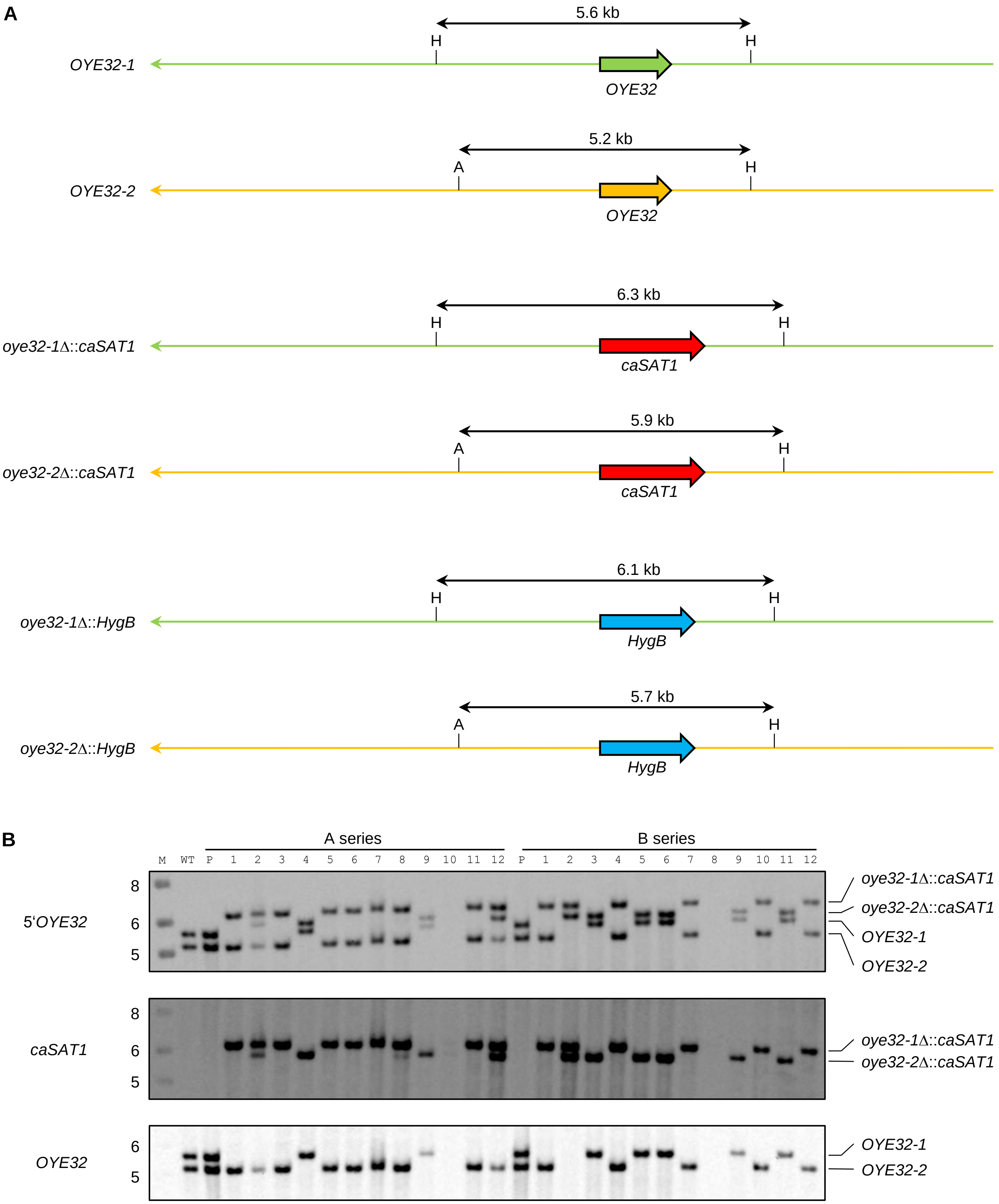

FIG S6 Deletion of *OYE32* using the *caSAT1* and *HygB* selection markers. (A) Structure of the *OYE32* locus in the wild type and in mutants containing the *caSAT1* and *HygB* selection markers in either of the two *OYE32* alleles. Arrows on the lines representing the chromosomes point towards the telomere. The locations of relevant AgeI [A] and HpaI [H] sites and the sizes of corresponding fragments are shown. (B) Southern hybridizations of AgeI/HpaI-digested genomic DNA of the wild-type strain SC5314 (WT), the parental strains SCMRR1R34A and SCMRR1R34B (P), and the two series of transformants derived from them after selection on plates containing nourseothricin with the probes specified on the left. The identities of the hybridizing fragments are indicated on the right side of the blots. M, size markers (in kb). Clones A10 and B8 could not be analyzed because of DNA loss. Continued on next page.

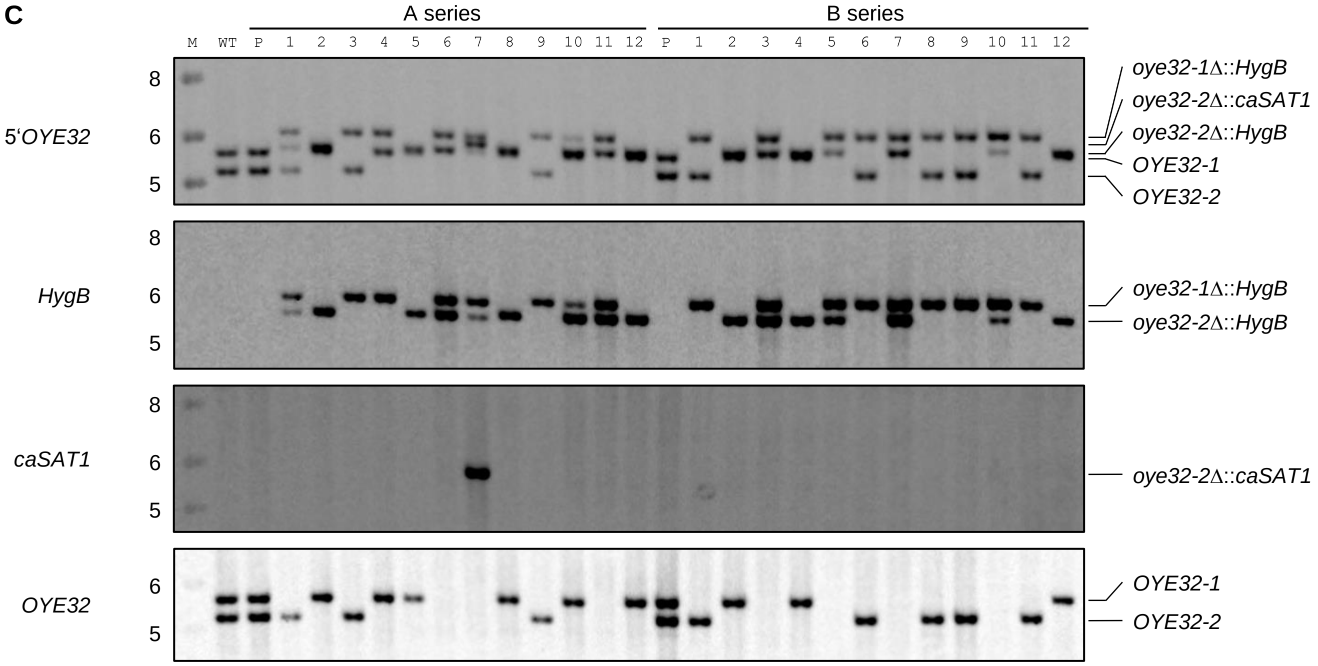

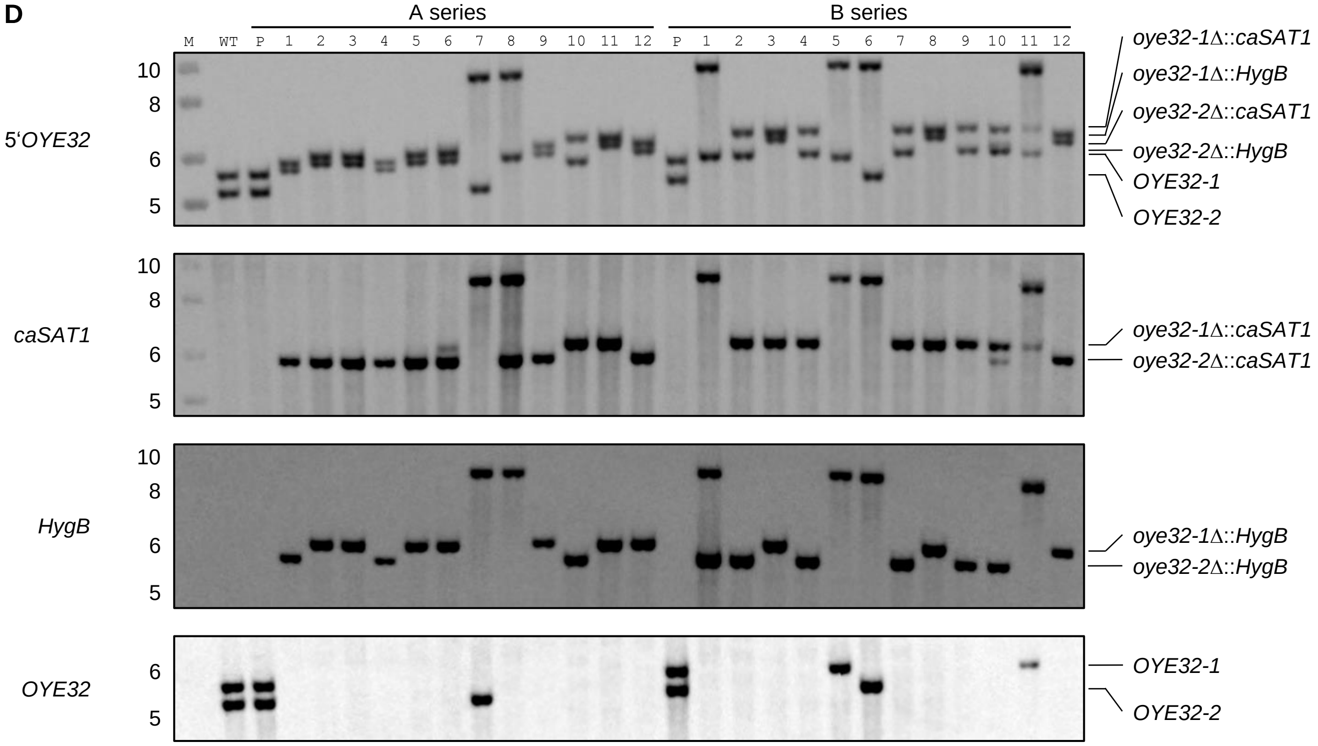

FIG S6 continued Southern hybridizations of AgeI/HpaI-digested genomic DNA of the wild-type strain SC5314 (WT), the parental strains SCMRR1R34A and SCMRR1R34B (P), and the two series of transformants derived from them after selection on plates containing hygromycin (C) or both nourseothricin and hygromycin (D) with the probes specified on the left. The identities of the hybridizing fragments are indicated on the right side of the blots. M, size markers (in kb). The size marker was inadvertently not included in the *HygB* probe in this experiment.
